# An assessment of implementation gaps and priority recommendations on food environment policies: the Healthy Food Environment Policy Index in Japan

**DOI:** 10.1017/S1368980021004900

**Published:** 2022-06

**Authors:** Miwa Yamaguchi, Marika Nomura, Yusuke Arai, Stefanie Vandevijvere, Boyd Swinburn, Nobuo Nishi

**Affiliations:** 1 International Center for Nutrition and Information, National Institute of Health and Nutrition, National Institutes of Biomedical Innovation, Health and Nutrition, 1-23-1 Toyama, Shinjuku-ku, Tokyo 162-8636, Japan; 2 Japan International Cooperation Agency, Tokyo, Japan; 3 Department of Nutrition, Chiba Prefectural University of Health Sciences, Chiba, Japan; 4 Public Health Nutrition Epidemiology and Public Health, Sciensano, Brussels, Belgium; 5 School of Population Health, University of Auckland, Auckland, New Zealand

**Keywords:** Food environments, Policy implementation, Priority actions, The Healthy Food Environment Policy Index

## Abstract

**Objective:**

The current study aimed to evaluate policies and actions for food environments by the Japanese Government using the Healthy Food Environment Policy Index (Food-EPI).

**Design:**

Public health experts rated the extent of implementation of food environment-related Policy and the Infrastructure-support components, compared with international best practices. Subsequently, the experts proposed and prioritised future actions to address implementation gaps in an online workshop.

**Setting:**

Japan.

**Participants:**

A total of sixty-six experts rated policy implementation by the Japanese Government and twenty-three participated in the workshop on future actions.

**Results:**

The implementations of regulations on unhealthy foods and non-alcoholic beverages were rated low in the domains of Food composition, Food labelling and Food promotion, Food prices and Food retail in the Policy component. The implementations of several domains in the Infrastructure-support component were, overall, rated at a higher level, specifically for monitoring and intelligence systems. Based on the rating, reducing health inequalities by supporting people, both economically and physically, was the highest priority for future actions in both components.

**Conclusions:**

The current study found that Japan has a robust system for long-term monitoring of population health but lacks regulations on unhealthy foods and non-alcoholic beverages compared with international best practices. The current study confirmed the importance of continuous accumulation of evidence through national monitoring systems. Developing comprehensive regulations to restrict food marketing, sales and accessibility of unhealthy foods and non-alcoholic beverages is needed to improve the health of food environments in Japan.

Non-communicable diseases (NCD) cause 41 million deaths each year, equivalent to 71 % of all deaths globally^(1)^. In Japan, the proportions of the population that are overweight (32·2 % in men and 21·9 % in women) and obese (4·3 % in men and 3·7 % in women)^(2)^ have remained stable for a decade^(3,4)^. The first, second and fourth leading causes of death in Japan are attributed to NCD^(5)^: malignant neoplasms, heart diseases and cerebrovascular diseases, respectively. Rates of NCD have seen a substantial increase over recent decades. The average salt intake in Japan is 10 g/d^(3)^, which is higher than the target (5 g/d) recommended by the WHO^(6)^. Considering these challenges, the second term of the National Health Promotion Movement in the 21st century (Health Japan 21 (the second term)^(7,8)^) is prioritising the prevention of NCD and extension of healthy life expectancy. There has been a call for action in Japan to put in place a social environment that supports and protects health^(7,8)^. Part of the actions is to create available and accessible food environments.

Food environments include the collective physical, economic, policy and socio-cultural aspects that influence people’s choices of foods and non-alcoholic beverages and their nutritional status^(9)^. Food environments are primarily influenced by the food industry, government and society^(9)^. The International Network for Food and Obesity Non-communicable Diseases Research, Monitoring and Action Support (INFORMAS)^(9)^ developed the Healthy Food Environment Policy Index (Food-EPI)^(10)^, which has been used in several countries as a validated tool and process, after some adaptations for a nation-specific context, for measuring policy implementation levels, to stimulate the implementation of policies that create healthy food environments to prevent obesity and NCD. The index comprised forty-seven common good practice indicators classified into thirteen domains: Food composition (COMP), Food labelling (LABEL), Food promotion (PROMO), Food provision (PROV), Food retail (RETAIL), Food prices (PRICES), Food trade and investment (TRADE) in the Policy component; and Leadership (LEAD), Governance (GOVER), Monitoring and intelligence (MONIT), Funding and resources (FUND), Platforms for interaction (PLATF) and Health-in-all policies (HIAP) in the Infrastructure-support component (Fig. 1). The major components of Food-EPI include collecting and verifying evidence of national action to create healthier food environments, rating implementation compared with the international best practices and proposing future actions for the government. These are done in collaboration with experts in the field.

**Fig. 1:**
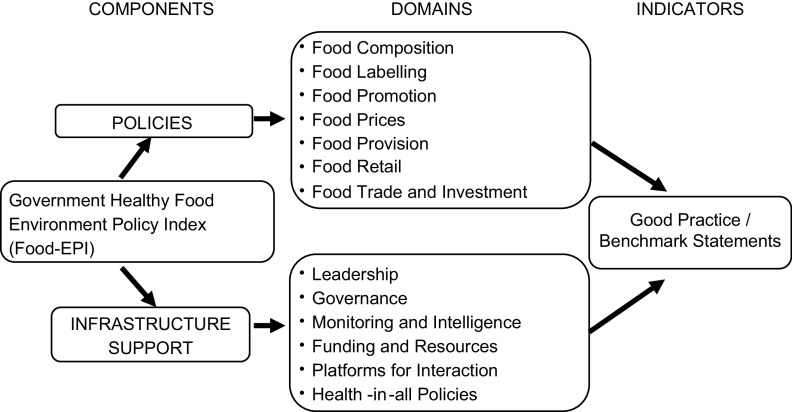
Components and domains of the Healthy Food Environment Policy Index (Food-EPI)

Previous studies of food-environment policies in Japan have some limitations. First, to our knowledge, no Japanese study has reported a comprehensive assessment of a national policy action that involves food environments. Although three reviews described history and current situation of Japanese food policies for health^(11)^, guidelines for dietary education^(12)^ and school nutrition programmes^(13)^, they did not assess the level to which these were implemented using a validated tool. Some Japanese studies investigated the associations between neighbourhood food environments and fruit and vegetable intake^(14,15)^, nutritional status^(16)^ and mortality^(17)^. However, those studies^(14–17)^ focused on the availability and/or accessibility of neighbourhood food environments and assessed the individual residents and not at the policy level. It is important to evaluate the implementation level of food policies to improve the health of food environments and prevent NCD. Second, there are no reports comparing the implementation level of Japanese policies with those of other countries using a standardised tool. A review article^(18)^ reported the implementation levels of food policies from 2015 to 2018 among eleven countries using the Food-EPI^(15)^. However, the review^(18)^ did not include any East Asian countries including Japan. The prevalence of obesity among adults in Japan (4·4 % in 2016) is comparatively lower than other WHO countries and regions^(4)^. Thus, an evaluation of food policies in Japan, a developed country in East Asia with low prevalence of obesity, could help to provide insight into these differences. To summarise and assess Japanese policies and actions using a globally standardised tool, the Food-EPI, makes it possible to understand the results comprehensively at the international level. Third, few studies evaluated the importance and feasibility of the future policies they proposed. It is important to evaluate the importance and feasibility of the proposed actions.

This study aimed to evaluate the policies and actions for food environments implemented by the Japanese Government using the Food-EPI.

## Methods

### Adaptation of the Food-Environment Policy Index protocol to the Japanese context

**Fig. 2:**
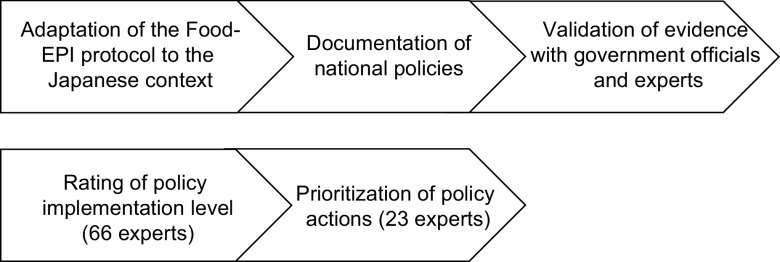
The protocol of the Healthy Food Environment Policy Index (Food-EPI) in Japan

In the first step (Fig. 2), we adapted the original protocol proposed by Swinburn et al.^(10)^ to the Japanese context without changing any domains and indicators in Food-EPI. Specifically, we changed the part of the process as follows: after the prioritisation of policy actions in the original protocol^(10)^, the next process was a ‘recommendations and translation of results for policymakers’ where the results of this survey were translated for the government and stakeholders to stimulate further policy implementation. However, we did not perform the process which would have required consultation with a government official. The Food-EPI domains and indicators were translated from English to Japanese. This process took approximately two months beginning April, 2019. Furthermore, an online workshop for the prioritisation of policy actions was conducted instead of the face-to-face workshop that was conducted in the original protocol^(10)^ given the COVID-19 outbreak.

### Documentation of national policies

For the second step shown in Fig. 2, we identified and reviewed policies proposed by the Japanese Government and their actions over the last three years (2016–2019), including long-term actions that were valid in 2019, in line with each indicator in the domain. This process was performed by one research representative and confirmed by three other researchers. We explored laws and public documents using the e-Gov database^(19)^, which contains the latest information. We explored related policies primarily through official websites, including the ‘Analysis and assessment project of the Health Japan 21 (the second term)’^(8)^ the aim of which was to describe the concrete targets and results of actions in relation to preventing NCD and extending healthy life expectancy; ‘Health, Medical Care’ from the website of Ministry of Health, Labour and Welfare^(20)^, which is a published document on the enforcement of health, diet, and nutrition education policies; and the websites of local governments in each prefecture. Other than official websites, we extracted the actions undertaken by non-governmental organisations when they were linked to policies. We summarised the evidence of implementation for each good practice indicator in the thirteen domains and then described the evidence in detail (see online supplementary material, Supplemental File 1).

Internationally recognised best practice policies for food environments were extracted from the NOURISHING framework adopted by the World Cancer Research Fund^(21)^ and the work of international experts from INFORMAS (2018). Best practice policies for food environments were then compared with the implementation of food policies in Japan (see online supplementary material, Supplemental File 1).

### Validation of evidence with government officials and experts

In the third step, four experts were contacted via e-mail to validate the evidence collected by the research team. Two experts belonged to a ministry and a local government, one expert belonged to an academic institution and had previously worked in a government organisation and one expert belonged to an international cooperation agency. All four experts confirmed the accuracy of the Japanese translation of the indicators. In addition, they corrected the description of policy actions and offered additional information to assess the degree of the policy implementation. Specifically, the experts recommended including relevant acts that the actions were based on (e.g. the Health Promotion Act^(22)^ for performing the Health Japan 21 (the second term)^(8)^ in the Leadership domain) and local community actions we overlooked (e.g. a training workshop for nutrition management in municipal governments in Food provision domain).

### Rating level of policy implementation

Rating level of policy implementation aims to investigate the degree to which the government has implemented a certain policy or action compared with international best practice exemplars (benchmarks). The degree of implementation considers the government’s intentions and plans, funding for implementation of actions and actions and policies partly or fully implemented and how well they are enforced^(10)^.

#### Participants in the rating of policy implementation

We recruited participants for the rating survey through the website of our institution. We recruited experts in public health nutrition and public health who were members of the following mailing lists: one academic conference (delivery number = 3204) and the related group of researchers (*n* 323), two networks for public health nutrition (*n* 471) and public health (*n* 1767), two research groups on social medicine and epidemiology (*n* 1110 and 45, including possible duplicate registrations with different e-mails), and one industrial network for public health nutrition (*n* 210). We requested experts to forward the recruitment notice to other networks or other experts they knew. The recruitment was conducted from November 12 to December 24, 2019.

Of the eighty experts who requested the research representative to send the detailed research plan by e-mail, seventy experts agreed to participate in the rating survey. We confirmed that all experts who agreed to participate in the current study had sufficient expertise through checking the research information of those via public databases and their affiliations.

#### Rating survey

By comparing international benchmarks as the 80–100 % implementation level, we asked experts to rate the implementation level of policies on a five-point Likert scale (i.e. < 20 %, 20–40 %, 40–60 %, 60–80 % and 80–100 %) for each indicator. The experts were requested not to consider whether the response would be generally accepted since the current study aimed to collect ratings based on the expert’s assessment. The evidence document we compiled was distributed to the experts from December 24 to 30 in 2019 – before the survey was initiated – so that they could review the Japanese policies beforehand. The experts responded with their ratings after confirming the summary of evidence and benchmarks in each indicator of the thirteen domains on the online survey. At the same time, they could refer back to the handout that was previously distributed if needed. In the online survey, the experts provided the following information: their affiliation (1. education and research institution, 2. government-related organisation including local government, 3. non-governmental organisation (NGO), 4. private institution of public health or 5. non-profit organisation (NPO); field of expertise (1. public health nutrition, 2. public health, 3. health and medical economy, 4. health and medical policy or 5. agriculture and fisheries) and years of experience in the specialty (1. < 3 years, 2. 3–4·9 years, 3. 5–9·9 years, or 4. ≥ 10 years). The experts responded ‘other’ if there were no appropriate selections. We conducted the rating survey online from January 6 to 29 February 2020. Of the seventy experts selected, sixty-six experts responded to the survey.

### Prioritisation of policy actions

After the rating survey, a workshop was organised for the experts to evaluate the challenges of the current food policies as identified from the ratings and to propose and prioritise concrete actions in the future. Their suggestions were considered to support future policy implementation of the government.

#### Participants

The agreement to participate in the online workshop of prioritisation of policy action was collected separately from that of the rating survey. Of the sixty-six experts, forty-one experts agreed to receive the invitation for the online workshop. Of which, twenty-three experts agreed to participate in the online workshop.

#### Prioritisation

The online workshop was held on 25 October 2020. A research representative first reported to the experts on the policy implementation gaps identified in the results of the rating survey. Group discussions (five to six experts per group) were conducted to propose concrete actions among indicators they regarded as important to improve healthy food environments and reduce obesity and diet-related NCD in Japan. When some groups proposed similar actions, we summarised those actions into one action. All future actions were decided through consensus from all experts.

On the second day of the online workshop, the research representative sent the sheet that listed the proposed future actions to the experts via e-mail and requested them to rank the actions from the highest to the lowest priority, taking into account the importance (i.e. the relative need, impact, effects on equity and any other positive or negative effects) and achievability (i.e. the relative feasibility, acceptability, affordability and efficiency) of each action, respectively^(23)^. The rankings that each expert provided were not shared with other experts to avoid influencing their views. All responses on the prioritisation of future actions were received by e-mail within a week.

### Data analyses

#### Rating of policy implementation level

To average the results, we replaced the response categories of < 20 %, 20–40 %, 40–60 %, 60–80 % and 80–100 % for policy implementation level with 20 %, 40 %, 60 %, 80 % and 100 %, respectively. Responses of ‘cannot rate’ were analysed as missing values. We then classified the implementation level into four categories: ‘very low if any’ (≤ 25 %), ‘low’ (26–50 %), ‘medium’ (51–75 %) and ‘high’ (> 75 %). The mean and sd in each domain were calculated as the sum of the implementation levels of the indicators divided by the number of responses. To investigate the level of agreement, Gwet’s AC^2^ inter-rater reliability (IRR) coefficient and 95 % CI were calculated for the overall results as well as the results for each Policy and Infrastructure-support component, and the two stakeholders (academia and government) using the Agreestat software (Agreestat 2013·1, Advanced Analytics).

#### Prioritisation of policy actions

We calculated the average ranking scores for the Policy and the Infrastructure-support components and plotted the importance and achievability of each on a quadrant graph to investigate the balance of actions. Actions in the top third for importance were selected as the highest priority for the implementation.

## Results

### Rating of policy implementation level

#### Characteristics of participants and the agreement level of the rating

**Fig. 3:**
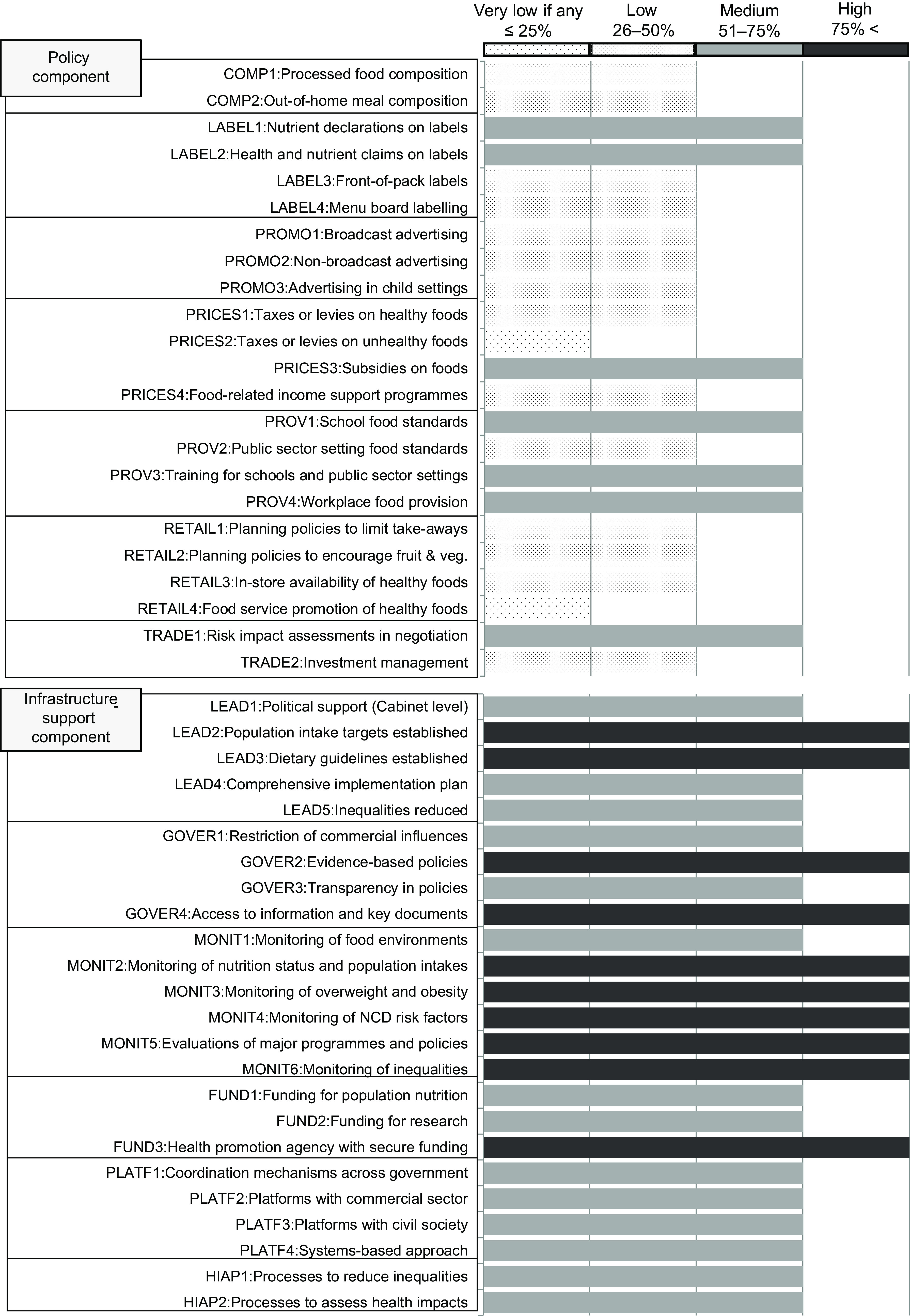
Implementation level of indicators against international best practices in the Policy component and the Infrastructure-support component. COMP, Food composition; LABEL, Food labelling; PROMO, Food promotion; PRICES, Food prices; PROV, Food provision; RETAIL, Food retail; TRADE, Food trade and investment; LEAD, Leadership; GOVER, Governance; MONIT, Monitoring and intelligence; FUND, Funding and resources; PLATF, Platforms for interaction; HIAP, Health-in-all policies. The inter-rater reliability (IRR) and 95 % confidential interval (CI) was 0·44 (95 % CI: 0·38, 0·50) in overall, 0·65 (95 % CI: 0·58, 0·72) the Policy component and 0·44 (95 % CI: 0·36, 0·52) the Infrastructure-support component. The IRR in the academia group was 0·45 (95 % CI: 0·38, 0·53) and 0·49 (95 % CI: 0·41, 0·58) in the government experts

Of the sixty-six experts, most of them belonged to educational and research institutions (66 %) or government institutions (21 %; Table 1). The specialties of most experts were related to public health nutrition (57·6 %) and public health (27·3 %), and 58·6 % of experts had over ten years of experience in their specialty. There were no experts who belonged to NGO or NPO. The average implementation levels compared with internationally recognised best practice indicators within each of the thirteen domains are shown in Fig. 3. The means (sd) of the implementation level in each domain and indicator are shown in the online supplementary material, Supplemental File 2. The IRR of the overall rating was 0·44 (95 % CI: 0·38, 0·50; Fig. 3). The IRR of the Policy component (0·65, 95 % CI: 0·58, 0·72) was higher than that of the Infrastructure-support component (0·44, 95 % CI: 0·36, 0·52). There was no difference in the IRR between the group from academia (0·45, 95 % CI: 0·38, 0·53) and the government experts (0·49, 95 % CI: 0·41, 0·58).

**Table 1:** Characteristics of experts participating in the rating survey and the prioritisation workshop

	Number	%
Rating of policy implementation level (*n* 66)		
Affiliation		
Educational and research institution	44	65·7
Government-related organisation (including local government)	14	21·2
Private institution of public health	5	7·6
Others	3	4·5
Expertise field		
Public health nutrition	38	57·6
Public health	18	27·3
Health and medical economy	1	1·5
Agriculture and fisheries	1	1·5
Others	8	12·1
Years of experience in their specialty		
< 3 years	6	9·1
3–4·9 years	4	6·1
5–9·9 years	18	27·3
≥ 10 years	38	57·6
Prioritisation of policy actions (*n* 23)		
Affiliation		
Educational and research institution	18	78·3
Government-related organisation (including local government)	4	17·4
Private institution of public health	1	4·4
Others	–	
Expertise field		
Public health nutrition	13	56·5
Public health	8	34·8
Health and medical economy	–	
Agriculture and fisheries	1	4·4
Others	1	4·4
Years of experience in their specialty		
< 3 years	1	4·4
3–4·9 years	1	4·4
5–9·9 years	6	26·1
≥ 10 years	15	65·2

#### Policy implementation level in the Policy component

All indicators in the domains of Food composition, Food promotion and Food retail in the Policy component showed low implementation levels. Part of the indicators in the domains of Food labelling, Food prices, Food provision and Food trade and investment showed medium implementation levels: LABEL1 (Nutrient declarations on labels), LABEL2 (Health and nutrient claims on labels), PRICES3 (Subsidies on foods), PROV1 (School food standards), PROV3 (Training for schools and public sector settings), PROV4 (Workplace food provision) and TRADE1 (Risk impact assessments in negotiation). However, the average implementation levels in all domains except for Food provision and Food trade and investment were low (see online supplementary material, Supplemental File 2).

Of four indicators of Food provision, only PROV2 (Public sector setting food standards) indicated the low implementation level. In PROV1 (School food standards), the actions of the provision of school lunch and food and nutrition education (*Shokuiku* in Japanese^(24)^) were applied (see online supplementary material, Supplemental File 1). In PROV3 (Training for schools and public sector settings), the action of support and training systems of food services for schools and Specific Food Service Facilities were included. In addition to the school lunch system, healthy food services in workplaces were encouraged in PROV4 (Workplace food provision). Although there were some guidance and supports for food service activities in public sector settings and policies for healthy food choice in PROV2 (Public sector setting food standards), no law or act had been enforced for the policy action to regulate the kinds of foods and non-alcoholic beverages provided at events, fund-raising, sales promotions and vending machines.

In Food trade and investment domain, one of the two indicators (TRADE2: Investment management) showed low level of implementation. In TRADE2, no law or act had been enforced to manage the investment and protect their regulatory capacity for public health and nutrition (see online supplementary material, Supplemental File 1).

#### Policy implementation level in the Infrastructure-support component

As a whole, the Infrastructure-support component had a higher implementation level than the Policy component. All indicators in the other six domains were medium or high. Specifically, five of the six indicators (MONIT2: Monitoring of nutrition status and population intakes, MONIT3: Monitoring of overweight and obesity, MONIT4: Monitoring of NCD risk factors, MONIT5: Evaluations of major programmes and policies and MONIT6: Monitoring of inequalities) in Monitoring and intelligence domain showed a high implementation level, higher than the other domains in both the Policy and the Infrastructure-support components.

In the MONIT1 indicator, nutritional components in the nutrients of concern (i.e. salt, fat, saturated fat, trans fat and added sugar) were not regularly monitored in a school-lunch menu. Regarding the regular monitoring of nutritional status and dietary intake among adults and children in MONIT2, the National Health and Nutrition Survey (NHNS)^(25)^ was applied. Related to the actions in MONIT3, there were regular health check-ups for school children and workers to monitor the prevalence of overweight and obesity among them. In MONIT4, the Patient survey^(26)^ and the Ordinance of Vital Statistics Survey^(27)^ were applied to monitor the prevalence of NCD risk factors and occurrence of main diet-related NCD. In MONIT5, the management cycle incorporating the major factors of plan, do, check and action was applied to assess its effectiveness and contribution towards achieving the nutrition and health plans’ goals, such as the Health Japan 21 (the second term)^(8)^. Regarding the actions in MONIT6, data monitoring, conducted by the Comprehensive Survey of Living Conditions^(28)^ and the NHNS^(25)^, was utilised to reduce health inequalities or health impacts in vulnerable populations and societal and economic determinants of health.

### Prioritisation of policy actions

#### Characteristics of experts and policy actions

**Fig. 4:**
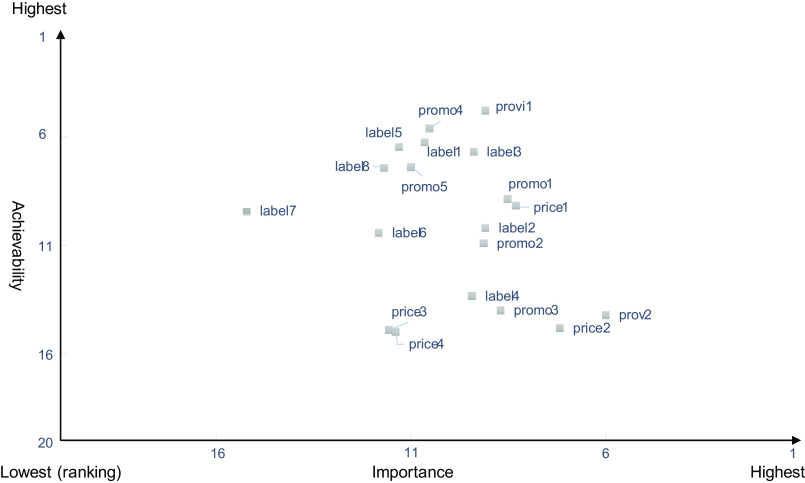
Priority of the importance and the achievability of policy actions in the Policy component

**Fig. 5:**
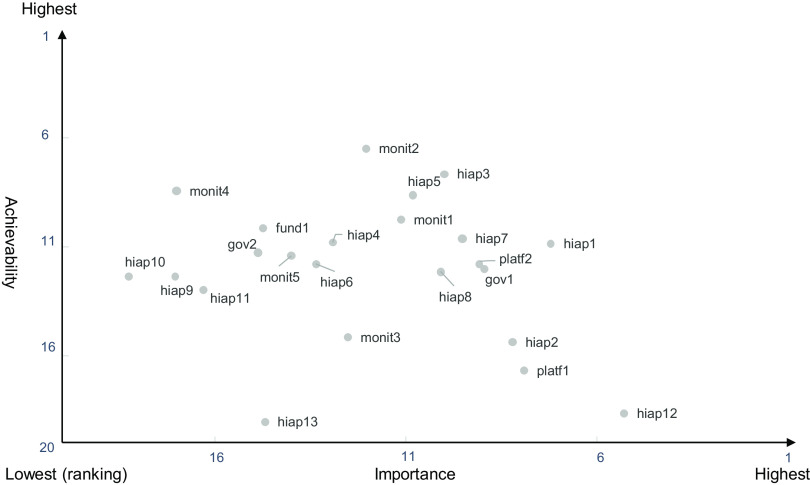
Priority of the importance and the achievability of policy actions in the Infrastructure-support component

Of the twenty-three experts who participated in the workshop, the proportion of those belonging to educational and research institutions (78·3 %) and with over ten years of experience in their specialty (65·2 %) was higher in the workshop than in the rating survey. The experts proposed nineteen actions across four domains within the Policy component, including Food labelling (eight actions), Food promotion (five actions), Food prices (four actions) and Food provision (two actions). The experts proposed twenty-three actions across the Infrastructure-support component, including five domains of Governance (two actions), Monitoring and intelligence (five actions), Funding and resources (one action), Platforms for interaction (two actions) and Health in all policies (thirteen actions) (Table 2). The quadrant graph indicated that the most important actions in both components had lower achievability compared with the other proposed actions (Fig. 4 and Fig. 5).

**Table 2:** The list of proposed priority in the policy and the infrastructure support actions

	**Policy**
	Food labelling
label1	Run media campaigns to educate consumers and raise their awareness about food labelling.
label2	Develop food labelling that even children can understand.
label3	Include food labelling in “*Shokuiku*” (Food and Nutrition Education) Promotion policies in order to incorporate food labelling into dietary education.
label4	Create a regulatory and monitoring system for food labelling and include it in the national budget.
label5	Education of people in the food industry about how to use the food labelling system by specialists from local private institutions and public health centres.
label6	Support small companies to make food labelling in compliance with the full enforcement of the new Food Labeling Act (April 2020).
label7	Construct a system for clinical nutrition counselling that utilises food labelling.
label8	Develop policies to raise the percentage of nutrition counselling that utilises food labelling (e.g. include the utilisation targets of food labelling into Health Japan 21 (the third term) and investigate the percentage of school lunch facilities that utilise food labelling).
	Food promotion
promo1	Encourage food industries to make self-standards for promoting healthy food consumption (e.g. create advertisements depicting eating vegetables with food products that contain high salt or sugar content, such as sauces and instant noodles) from the perspectives of SDG (Sustainable Development Goals) and ESG (Environment, Society, Governance).
promo2	Define unhealthy foods in order to raise public awareness of the necessity of policies for regulating unhealthy foods.
promo3	Regulate advertisements and call attention to advertisements for unhealthy foods.
promo4	Include health education about unhealthy foods targeted at children in food and nutrition education, as well as in the curriculum of health and physical education courses in schools.
promo5	Make opportunities to correctly educate the general public (adults) about unhealthy foods targeted at children in order to disseminate this information to people in all life stages.

	Food prices
price1	Accumulate scientific evidence regarding food prices in Japan (e.g. effect of taxation and tax reduction) to show the necessity of securing financial resources for taking policy actions.
price2	Raise consumption of fruits and vegetables through the taxation of unhealthy foods while simultaneously providing healthy foods at low prices to increase the accessibility of vegetables and fruits.
price3	Prioritise taxation of foods containing high amounts of sugar more than ones that contain high amounts of trans and saturated fats to prevent dental caries and obesity among children.
price4	Provide economic support for agricultural producers when the government lowers the price of vegetables and fruits.
	Food provision
prov1	Promote food and nutrition education (including school lunch) especially for children in preschool and compulsory education (elementary and junior high school) so that they can learn how to make healthier food choices.
prov2	Strengthen Japanese policies regarding enhancing the environments surrounding food system and provision, such as ensuring that the salt content in bread is lower to nudge people who are not interested in reducing their salt intake towards making healthier choices.

	**Infrastructure support**
	Governance
gov1	Build a system that accommodates claims from restaurants, food companies and other related stakeholders when implementing policies related to topics such as taxation and regulation of unhealthy food.
gov2	Improve the system of nutrition claims about food, such as Foods with Nutrient Function Claims (FNFC), to make it easier to understand, in order to nudge people to make a healthier choice.
	Monitoring and intelligence
monit1	Review the way in which the National Health and Nutrition Surveys are conducted to improve the response rate. For example, utilise the Specific Health Checkups and Specific Health Guidance database to monitor and conduct online surveys.
monit2	In addition to gathering and monitoring new data, analyse and study trends from previous national survey data.
monit3	Perform cohort studies at the national level based on the monitoring data and analyse and research any individual-level variation.
monit4	Analyse the monitoring data and investigate the gaps between the national targets and the results of the national survey conducted by several specialists with various areas of expertise.
monit5	With government support, collect varied and diverse monitoring data.
	Funding and resources
fund1	Establish national research grants that specialise in research topics on unhealthy foods and health effects (e.g. research on sugar and dental caries) in order to collect important evidence for policy actions.
	Platforms for interaction
platf1	Establish a department to improve food environments in the local governments of all prefectures.
platf2	In cooperation with the government and food industries, create guidelines for choosing between healthy and unhealthy foods.

	Health-in-all policies
hiap1	Perform an industry-academia-government collaboration, such as with the Ministry of Agriculture, Forestry and Fisheries, Ministry of the Environment, and Ministry of Economy, Trade and Industry, to further proceed with health and food environmental policies.
hiap2	Enhance a multi-disciplinary approach such as an obligation to assign registered dietitians who could introduce their insight about healthy diet and nutrition into policies in the departments where there are no nutritional specialists.
hiap3	Determine the current status of factors related to health disparities in the field of food and nutrition.
hiap4	Strengthen policies (e.g. free canteens for children called "*Kodomo Shokudou*" in Japanese) that may help to narrow income gaps that lead to health disparities.
hiap5	Decide on policies related to the health and food environment after determining which policies should get priority, in accordance with various research and survey reports.
hiap6	Provide education and social support for children who are not very aware of the importance of health, in order to reduce health disparities.
hiap7	Be sure to collaborate with experts and stakeholders who acknowledge the importance of food and nutrition when formulating policies about healthy food environments.
hiap8	Accommodate a food and nutrition, as well as health perspective, into policies such as economic, agricultural and environmental policies.
hiap9	Take measures to determine targets for each business establishment for health management (“*Kenko Keiei*” in Japanese), which is a management strategy to raise productivity by keeping and improving the health of employees.
hiap10	Demonstrate the economic benefits of providing specific health guidance.
hiap11	Establish a national certification system for health management practices (“*Kenko Keiei*”) implemented by companies.
hiap12	Develop a comprehensive law, such as the Food Environment Improvement Promotion Law (tentative), to improve food environments.
hiap13	Propose strategies to WHO that promote comprehensive healthy food environments, including industry-academia-government collaborations, so that they can be shared with other countries.

#### Prioritised policy actions in the Policy component

The top three policy actions in terms of importance were as follows: (1) (prov2) in Food provision domain, ‘strengthen Japanese policies regarding enhancing the environments surrounding food system and provision, such as ensuring that the salt content in bread is lower to nudge people who are not interested in reducing their salt intake towards making healthier choices'; (2) (price2) in Food prices domain, ‘raise consumption of fruits and vegetables through the taxation of unhealthy foods while simultaneously providing healthy foods at low prices to increase the accessibility of vegetables and fruits’ and (3) (price1), ‘accumulate scientific evidence regarding food prices in Japan (e.g. effect of taxation and tax reduction) to show the necessity of securing financial resources for taking policy actions’.

#### Prioritised policy actions in the Infrastructure-support component

In the Infrastructure-support component, the following actions were prioritised, based on their importance, as the top three: (1) (hiap12) regarding Health-in-all policies domain, ‘develop a comprehensive law, such as the Food Environment Improvement Promotion Law (tentative) to improve food environments’; (2) (hiap1), ‘perform an industry-academia-government collaboration, such as with the Ministry of Agriculture, Forestry and Fisheries, Ministry of the Environment, and Ministry of Economy, Trade and Industry, to further proceed with health and food environmental policies’ and (3) (platf1) in Platforms for interaction domain, ‘establish a department to improve food environments in the local governments of all prefectures.’

## Discussion

The current study is the first to implement the Food-EPI in Japan, an East Asian country, and provides important insight into the extent to which internationally recommended policies for healthier food environments have been implemented by the Japanese Government. The findings indicate that there are very few policies that regulate unhealthy food and support healthier food environments in Japan, with majority of the indicators under the Policy component rated as low implementation. The Infrastructure-support component performed better than the Policy component, with a number of indicators rated as medium and high implementation. In the future, developing comprehensive regulations on unhealthy foods and non-alcoholic beverages should be prioritised to support socially vulnerable people in making healthier choices and reduce health inequalities.

### Rating of policy implementation level

#### The agreement level of the rating

The IRR of 0·44 in the present study was lower than the 0·60 to 0·82 range reported in eleven other countries^(18)^, although the IRR (0·65) of the Policy component in the current study was similar to that in other countries. The experts were more likely to have different perspectives on the implementation level of the Infrastructure-support component in Japan than those in other countries.

#### Policy implementation level in the Policy component

In the Policy component, the proportion (about 70 %) of indicators with lower implementation levels in the present study was higher than that of Singapore, Chile and Australia^(18)^. Whereas the present trend of low implementation of domains in the Policy component was relatively close to that of Malaysia, Canada, New Zealand and England^(18)^. Specifically, the low implementation level of indicators in the domain of Food composition in Malaysia^(18)^ was in line with the result in the current study. The results of the present study related to the low implementation level of policies related to Food promotion and Food retail were also consistent with Malaysia, Canada, New Zealand and England^(18)^. It is possible to develop actions that are not only based on national policies but also on common global goals, such as the seventeen Sustainable Development Goals^(29)^ and Nutrition for Growth^(30)^. The low implementation level in the Policy component in Japan can be characterised as the lack of regulations and restrictions on unhealthy foods and non-alcoholic beverages especially in Food composition, Food labelling, Food promotion, Food prices and Food retail in settings of schools, public sectors, retailers and community events. It is likely that there are several factors that have contributed to low levels of implementation of policies related to healthier food environments. Nevertheless, two possible factors could be mentioned. First, the government has focused on nutrition education, *Shokuiku* ^(24)^, in schools as a way to encourage people to have healthier diets and food choices from childhood, rather than focusing on regulatory interventions. To establish a new regulatory system for unhealthy food in Japan, further studies, such as simulation studies and intervention studies, are required to find an effective and feasible way of regulation. Second, the low prevalence of obesity in Japan^(4)^ may have reduced the urgent necessity of regulations on unhealthy food. Nutrition education may have caused people to be conscious of their health, which may have contributed to the low prevalence of obesity in Japan. According to the Third Basic Program for *Shokuiku* Promotion^(24)^, the *Shokuiku* Promotion was aimed to prevent obesity not only among children but also among adults.

It was notable that most of the indicators (three out of four) of Food provision domain in the Policy component received a medium rating for the level of implementation. Since the end of World War II (at the beginning of 1946)^(24,31)^, Japan has provided nutritious school lunches and food and nutrition education (*Shokuiku*) as a part of the health education. The national survey in 2019^(32)^ showed that 95·2 % of schools of mandatory education (from 6 to 15 years old) provided school lunches. This action of providing healthy food to school children might contribute to the medium implementation level in Food provision. However, the domain of Food provision overall failed to reach a high implementation level. The low implementation level of the PROV2 indicator suggests that the lack of action related to healthy food provision for children in public settings other than schools (i.e. food products at events, sales promotions and vending machines) might have prevented the domain from reaching beyond the medium implementation level.

#### Policy implementation level in the Infrastructure-support component

Interestingly, in the Infrastructure-support component, there were no indicators with low implementation level in the current study. Especially in the domain of Monitoring and intelligence, five of the six indicators showed a high implementation level. Although about 80 % of all indicators had a medium or high implementation level in Singapore, no country reported medium or high implementation levels across all the Infrastructure-support indicators^(18)^. The high performance in Monitoring and intelligence domain may be reflective of the Japanese health monitoring systems. The NHNS, which is conducted annually in Japan, originally began as the National Nutrition Survey in 1945 after World War II^(25)^ and is currently conducted by the Health Promotion Act^(22)^. There has been also a system to monitor mortality from NCD since 1899^(27)^, and annual health check-ups for children at schools since 1958^(33)^ and workers at workplaces since 1972^(34)^.

### Prioritisation of policy actions

#### The characteristics of the highest prioritised actions

The overall aim of the highest prioritised actions was to reduce health inequalities targeting socially vulnerable people in both the Policy and Infrastructure-support components. The actions in the Policy component were proposed to encourage and guide people to make healthy choices. Furthermore, to promote actions in the Policy component, actions extending healthy life expectancy and reducing health disparities in the Infrastructure-support component were proposed, and that was consistent with the national goal in Health Japan 21 (the second term)^(8)^. In the NHNS for fiscal year 2019^(29)^, a specific questionnaire was added to investigate the establishment of a social environment where people can naturally improve their health. One of four citizens responded that they did not intend to improve their dietary habits or increase physical exercise according to the results of the NHNS^(29)^.

#### The relationship between the Policy and the Infrastructure-support components

The following actions, among those of the highest priority, were categorised as having low achievability: enhancing the environments surrounding food system and provision to reduce salt intake without an individual conscientiously trying to do so (prov2 in Food labelling of the Policy component) and developing a comprehensive law to improve food environments (hiap12 in Health-in-all policies of the Infrastructure-support component). Experts pointed out several challenges associated with implementing the identified priority actions in Japan. First, it will take a long time to enhance policies and systems related to the actions of prov2 and hiap12. The top three priorities in terms of importance in the Policy component were linked to the second and third priority in the Infrastructure-support component. When we build an education system to promote healthy food choices among children at an early age in the Policy component, local governments are required to create platforms for this, which is part of the Infrastructure-support component. In addition, when we aim to impose taxation on unhealthy food and reduce the price of healthy food in the Policy component, an industry-academia-government collaboration is needed in the Infrastructure-support component. The present result showed that the most important action was to establish a comprehensive law on healthy food environments in the Infrastructure-support component. The result clarified that most experts recognise the importance of developing a comprehensive law from several aspects related to the regulations on food marketing, sales and accessibility.

### Strengths and limitations

The strength of the current study includes its use of an internationally validated index and tool that have been applied in eleven other countries and is based on a rigorous methodology with a long consultation^(18)^. The information regarding the policies and actions we employed was reliable since it was confirmed by government officials and experts who had sufficient knowledge about Japanese policies and actions. In addition, the assessment of policy implementation level by experts with sufficient expertise and knowledge was legitimate. Despite these strengths, our study has some limitations that deserve mention. First, some selection bias may impede reproducibility as the experts volunteered to participate in the present rating survey and workshop. There may be some experts who were unable to participate in the rating survey and workshop due to scheduling conflicts. Furthermore, there might be a bias related to the expertise of the experts who participated in this survey, because it was difficult to appropriately allocate them to each domain that matched their expertise. Second, the level of agreement among experts on policy implementation, especially in the Infrastructure-support component, was low according to the IRR in the current study. We should note the existence of varying perspectives on the current policies, which is the reason behind the present results. Third, the results of the current study only provide a snapshot of the current practice in Japan. Continuous monitoring and assessment of government policies and practices in Japan are required to track the progress over time and increase government accountability related to healthy food environments, similar to what the study in New Zealand performed as a second investigation^(35)^. Finally, there may have been some time-related discrepancies between the results of the rating survey and the policy prioritisation workshop because they were conducted nine months apart. To the best of our knowledge, the only policy change to occur during this time was an update to the Food Labeling Act (April 2020)^(36)^, which saw minor changes to the requirements for the labelling of allergens, energy and nutritional values.

### Conclusion

The current study found that Japan has a robust system – the NHNS – for the long-term monitoring of population health. However, it lacks regulations on food marketing, sales and the accessibility of unhealthy foods and non-alcoholic beverages. Therefore, stronger regulations to restrict unhealthy foods and non-alcoholic beverages, along with regular monitoring of government progress, need to be prioritised in future policy actions to establish comprehensive healthy food environments.

## Supplementary material

For supplementary material accompanying this paper visit https://doi.org/10.1017/S1368980021004900. click here to view supplementary material
