# Supplementary material for: An assessment of implementation gaps and priority recommendations on food environment policies: the Healthy Food Environment Policy Index in Japan
Source: Public Health Nutr. 2021 Dec 20;25(6):1720–32. doi: 10.1017/S1368980021004900 (PMC9991651; doi:10.1017/S1368980021004900)
Supplement: Supplementary file 1 [file S1368980021004900sup.zip › S1368980021004900sup001.docx]

Supplemental File 1: The good practice of policies and actions of food environments in Japan

**Policy component**

| **1 FOOD COMPOSITION**  There are government systems implemented to ensure that, where practicable, processed foods minimise the energy density and the nutrients of concern (salt, fat, saturated fat, trans fat, added sugar) |
| --- |
| **COMP 1**: Food composition targets/standards have been established for processed foods by the government for the content of the nutrients of concern in certain foods or food groups if they are major contributors to population intakes of these nutrients of concern (trans fats and added sugars in processed foods, salt in bread, saturated fat in commercial frying fats) |
| <Laws/Acts>  No law/act has been formulated which specifies certain (processed) foods that contain nutrients of concern, and which shows a target amount/standard value of those food components, nor have such policies have been formulated.  Supplementary comments  1) In Article 2, Item Ro-2 under Article 16 of the Health Promotion Act, it is stated that an excessive intake of fat, saturated fat, cholesterol, sugar and sodium equivalent to salt, prevents the promotion of health with showing the Dietary Reference Intakes for Japanese. However, there is no target or standard amount given for food or food groups on nutrients of concern.  2) Based on the result of the National Health and Nutrition Survey, it was found that the average intake of trans fat per day per person was comparatively small amount. |
| **International best practice benchmark(s)**  ARGENTINA: In December 2013, the Government adopted a law on mandatory maximum levels of sodium permitted in meat products and their derivatives, breads and farinaceous products, soups, seasoning mixes and tinned foods (Act 26905) which entered into force in December 2014. The law is applicable to salt levels in restaurant dishes. Infringements by producers and importers may be sanctioned, the most severe penalties being fines of up to one million pesos, in case of repeat infringements up to ten million pesos, and the closing of the business for up to five years.  SOUTH-AFRICA: In 2013, the South African Department of Health adopted mandatory targets for salt reduction in 13 food categories (including bread, breakfast cereals, margarines and fat spreads, savory snacks, processed meats as well as raw-processed meat sausages, dry soup and gravy powders and stock cubes) by means of regulation (Foodstuffs, Cosmetics and Disinfectants Act).  DENMARK: A law introduced in 2003 prohibits the sale of products containing trans-fats, a move that effectively bans its use in products destined for sale on the Danish market. The law is enforced by local authorities under the supervision of the Danish Veterinary and Food Administration. Infringement of the law may incur a fine or imprisonment, and companies can be prosecuted according to the Danish Penal Code.  EU & UK: In 2012, under the directive 2012/12/EU of The European Parliament and the Council, an amendment of Council Directive 2001/112/EC outlined that addition of sugars is no longer authorised in fruit juice. Similarly, added sugar in fruit juice is no longer permitted under The Fruit Juices and Fruit Nectars Regulations 2013. |
| **COMP 2**: Food composition targets/standards have been established for out-of-home meals in food service outlets by the government for the content of the nutrients of concern in certain foods or food groups if they are major contributors to population intakes of these nutrients of concern (trans fats, added sugars, salt, saturated fat) |
| <Laws/Acts>  No law/act has been formulated on the target or standard amount of nutrients of concern for out-of-home meals provided at food service outlets.  Supplementary Comments  In Health Promotion Act, it is stated that in view of nutrient intake, an excessive intake of fat, saturated fat, cholesterol, sugar and sodium equivalent to salt, prevents health among nationals, and these reference intakes are shown by Dietary Reference Intakes for Japanese.  <Policies>  “Healthy Diet” that supports the longevity of Japanese people proposed by the Ministry of Health, Labour and Welfare, shows the standard amount of energy and salt in foods supplied in out-of-home meals. However, no standard is set for fat, saturated fat, trans fat and sugar. |
| **International best practice benchmark(s)**  ARGENTINA: In December 2013, the Government adopted a law on mandatory maximum levels of sodium permitted in meat products and their derivatives, breads and farinaceous products, soups, seasoning mixes and tinned foods (Act 26905) which entered into force in December 2014. The law is applicable to salt levels in restaurant dishes. The legislation is embedded into a wider initiative (Less Salt, More Life) which also includes the reduction of salt in processed foods through voluntary agreements with manufacturers, retailers and bakers, and public awareness of the health effects and the need to reduce discretional salt.  US: In June 2015, the US Food and Drug Administration determined that partially hydrogenated oils (PHOs), the primary source of trans fats, are not “generally recognized as safe (GRAS)” for any use in food. Food manufacturers had three yeas to remove them from products. As of 18 June 2018, food manufacturers and restaurants are no longer allowed to produce foods that contain PHOs. |
| **2 FOOD LABELLING**:  There is a regulatory system implemented by the government for consumer-oriented labelling on food packaging and menu boards in restaurants to enable consumers to easily make informed food choices and to prevent misleading claims |
| **LABEL1**: Ingredient lists and nutrient declarations in line with Codex recommendations are present on the labels of all packaged foods |
| <Laws/Acts>  The Food Labeling Act stipulates that the name of the product, allergens, method of preservation, expiry date, raw materials, additives, amount of nutrition components and calories, product of origin and other necessary information required should be clearly stated on the food label for sale. In addition, the method of description and other necessary items for food suppliers to comply with, in describing the aforementioned items are also stipulated.  <Policies>  1) Generally, labelling of raw materials and nutrients of foods is based on Food Labeling Act in Japan. Therefore, international food standards of Codex Alimentarius Commission on labelling are not necessarily applied to all packaged foods in Japan.  2) Since the inception of the Codex Consultation Meeting in 2000, various discussions have been made continuously so that Japanese packaging should comply with international food standards of Codex Alimentarius Commission. |
| **International best practice benchmark(s)**  MANY COUNTRIES: In a wide range of countries producers and retailers are required by law to provide a comprehensive nutrient list on pre-packaged food products (with limited exceptions), even in the absence of a nutrition or health claim. The rules define which nutrients must be listed and on what basis (e.g., per 100g/per serving). |
| **LABEL2**: Robust, evidence-based regulatory systems are in place for approving/reviewing claims on foods, so that consumers are protected against unsubstantiated and misleading nutrition and health claims |
| <Laws/Acts>  The Food Labeling Standards, as stipulated in Item 1 of Article 4, Food Labeling Act, has been enforced as of 1^st^ April 2015. This Act makes it a rule to describe food nutrition on packaged foods, and hence, all processed foods and additives for general use, in principle, have been regulated to show nutrition components on the package.  <Policies>  There are regulatory systems in place that prevent claims or advertisement on nutrients and health effects that are not science-based or misleading by the following measures. Regulating the description of Food for Specified Health Uses that are to be examined individually by the Secretary General of the Consumer Affairs Agency. And indicating the guideline that a food industry should comply with as they submit to the authority of Foods with Functional Claims for specific health objectives. |
| **International best practice benchmark(s)**  AUSTRALIA/NEW ZEALAND: A law (Standard 1.2.7), approved in 2013, regulates the use of nutrition content and health claims on food labels in Australia and New Zealand. Health claims must be based on pre-approved food-health relationships or self-substantiated according to government requirements and they are only permitted on foods that meet nutritional criteria, as defined by a nutrient profiling model (Nutrient Profiling Scoring Criterion (NPSC)) taking into account energy, sodium, saturated fat and total sugar content of foods, as well as protein, fibre, fruit, vegetable, nut and legume content of foods.  INDONESIA: Regulation HK.03.1.23.11.11.09909 (2011) on "The Control of Claims on Processed Food Labelling and Advertisements" establishes rules on the use of specified nutrient content claims (i.e., levels of fat for a low-fat claim). The Regulation applies to any food product or beverage which has been processed. Generally, any nutrition or health claim may only be used on processed foods or beverages if they do not exceed a certain level of fat, saturated fat and sodium per serving (13g total fat, 4g saturated fat, 60mg cholesterol and 480mg sodium).  US: Nutrient-content claims are generally limited to a list of nutrients authorized by the Food and Drug Administration (Food Labelling Guide 1994, as last revised in January 2013). Packages containing a nutrient-content claim must include a disclosure statement if a serving of food contains more than 13g of fat, 4g of saturated fat, 60mg of cholesterol, or 480mg of sodium. Health claims are generally not permitted if a food contains more than 13g of fat, 4g of saturated fat, 60mg of cholesterol, or 480mg of sodium. Sugar and whole grain content are not considered. |
| **LABEL3**: A single, consistent, interpretive, evidence-informed front-of-pack supplementary nutrition information system, which readily allows consumers to assess a product’s healthiness, is applied to all packaged foods |
| <Laws/Acts> and <Policies>  No law/act and policies have been formulated that regulates front-of-pack labelling. |
| **International best practice benchmark(s)**  AUSTRALIA/NEW ZEALAND: The government approved a 'Health Star Rating' (HSR) system as a voluntary scheme for industry adoption. The system takes into account four aspects of a food associated with increasing risk for chronic diseases; energy, saturated fat, sodium and total sugars content along with certain 'positive' aspects of a food such as fruit and vegetable content, and in some instances, dietary fibre and protein content. Star ratings range from ½ star (least healthy) to 5 stars (most healthy). The Technical Advisory Group is currently evaluating progress as well as conducting a formal review of the HSR system, including an assessment of the underlying algorithm. In New Zealand, as of March 2016, about 900 products have stars on them (March 2016).  UK: Traffic light labelling has been recommended for use in the UK since 2006. In 2013, the Government published national guidance for voluntary 'traffic light' labelling for use on the front of pre-packaged food products. The label uses green, amber and red to identify whether products contain low, medium or high levels of energy, fat, saturated fat, salt and sugar. The format of the label and thresholds for nutrients of concern for red, amber and green can be found elsewhere.  CHILE: In 2012, the Chilean Government approved a Law of Nutritional Composition of Food and Advertising (Ley 20, 606). In June 2015, the Chilean authority approved the regulatory norms required for the law’s implementation (Diario Oficial No 41.193). The regulatory norms define limits for calories (275 calories/100g or 70 calories/100mL), saturated fat (4g/100g or 3g/100mL), sugar (10g/100g or 5g/100mL) and sodium (400mg/100g or 100mg/100mL) content considered “high” in foods and beverages. All foods that exceed these limits need to have a front-of-package black and white warning message inside a stop sign that reads “HIGH IN” followed by CALORIES, SATURATED FAT, SUGAR or SODIUM, as well as “Ministry of Health”. |
| **LABEL4**: A consistent, single, simple, clearly-visible system of labelling the menu boards of all quick service restaurants (i.e. fast food chains) is applied by the government, which allows consumers to interpret the nutrient quality and energy content of foods and meals on sale |
| <Laws/Acts> and <Policies>  No law/act and policies been formulated that regulates the description of nutrition components on the menu boards of fast-food chains.  Supplementary Comments:  1) The government shows energy amounts contained in fast-food meals via the health information site “e-health net” so that it could inform consumers of useful information to prevent non-communicable diseases.  2) Fast-food chains show nutrition components contained in their meals on sale on a voluntary basis. |
| **International best practice benchmark(s)**  AUSTRALIA: Legislation in Australian Capital Territory (Food Regulation 2002) and the States of New South Wales (Food Regulation 2010), South Australia (Food Regulation 2002) and Queensland (Food regulation March 2017) requires restaurant chains (e.g., fast food chains, ice cream bars) with ≥20 outlets in the state (or seven in the case of ACT), or 50 or more across Australia, to display the kilojoule content of food products on their menu boards. Average adult daily energy intake of 8700kJ must also be prominently featured. Other chains/food outlets are allowed to provide this information on a voluntary basis, but must follow the provisions of the legislation.  SOUTH KOREA: Since 2010, the Special Act on Safety Control of Children’s Dietary Life has required all chain restaurants with 100 or more establishments to display nutrient information on menus including energy, total sugars, protein, saturated fat and sodium.  TAIWAN: Since July 2015, convenience store chains, drink vendor chains and fast-food chains have to label the sugar and caffeine content of prepared-when-ordered drinks (e.g., coffee-and-tea-based drinks, fruit and vegetable juices) according to a regulation based on the Food Safety and Sanitation Act. The amount of sugar added to drinks (specified in sugar cubes) and its calorie content have to be displayed on drink menus and/or notice boards in a prescribed minimum font. In addition, different colours have to be used to signal the level of caffeine contained in coffee drinks. |
| **3 FOOD PROMOTION**:  There is a comprehensive policy implemented by the government to reduce the impact (exposure and power) of promotion of unhealthy foods to children (<16years) across all media |
| **PROMO1**: Effective policies are implemented by the government to restrict exposure and power of promotion of unhealthy foods to children through broadcast media (TV, radio) |
| <Laws/Acts>  No law/act has been formulated.  Supplementary Comments:  The Health Promotion Act and Basic Act on Consumer Policies both prohibit overstated expressions on advertisements, and also related descriptions; however, they are targeted to the whole nationals, not specifically to children.  <Policies>  1) In the symposium to explore advertisements towards children sponsored by the Prime Minister’s Office, discussions were made not only on the advertisement of foods but on the methods of advertisements targeted to children by various media, including broadcasting.  2) The broadcasting standards of the Japan Commercial Broadcasters Association stipulate that such advertisements should be well considered to suit children. |
| **International best practice benchmark(s)**  NORWAY: Under the Broadcasting Act of 1992 (Chapter 3.1), Norway prohibits marketing directed at children under 18 and advertising in connection with children’s programmes on TV, radio and teletext. The ban includes any product, including food and beverages, but only applies to broadcast media originating in Norway. The Broadcasting Regulation No 153 of 1997 provides guidance on how to determine whether advertising is directed to children under 18: whether the advertised product or service is of particular interest to children, if animation or other forms of presentation are used that particularly appeal to children, the time the advertisement is broadcast, and whether children under the age of 13 appear in the advertisement.  IRELAND: Advertising, sponsorship, teleshopping and product placement of foods high in fats, sugars and salt, as defined by a nutrient profiling model, are prohibited during children’s TV and radio programmes where over 50% of the audience are under 18 years old (Children’s Commercial Communications Code, 2013 revision). In addition, there is an overall limit on advertising of foods high in fats, sugars and salt adverts at any time of day to no more than 25% of sold advertising time and to only one in four advertisements. Remaining advertising targeted at children under the age of 13 must not include nutrient or health claims or include licensed characters.  SOUTH KOREA: TV advertising to children less than 18 years of age is prohibited for specific categories of food before, during and after programmes shown between 5-7pm and during other children’s programmes (Article 10 of the Special Act on the Safety Management of Children’s Dietary Life, as amended 2010). |
| **PROMO2**: Effective policies are implemented by the government to restrict exposure and power of promotion of unhealthy foods to children through non-broadcast media (e.g. Internet, social media, food packaging, sponsorship, outdoor advertising including around schools) |
| <Laws/Acts>  No law/act has been formulated.  Supplementary Comments:  1) The Health Promotion Act restricts using expressions that contain unusually false facts or misleading nuances on the effects of health promotion or other items stipulated by the Cabinet Office Ordinance, when food-related suppliers advertise commercial foods.  2) The Act against Unjustifiable Premiums and Misleading Representations restricts or prohibits those actions that might hinder consumers’ choices, so that they are not attracted to the aforementioned.  3) The Act on Japanese Agricultural Standards (JAS Act) stipulates appropriate and rational standards for agricultural/forestry materials and appropriate expressions on the quality of agricultural/forestry materials, other than foods and beverages. By the Act, in conjunction with the measures provided by the Act on the Description of Food Contents, contributes to the smooth production and distribution of agricultural/forestry materials, the promotion of agricultural production based on needs and the protection of benefits of consumer.  4) The Health Promotion Act, Act against Unjustifiable Premiums and Misleading Representations and JAS Act are targeted not only to children but to the whole nationals.  5) In the Act on Development of an Environment that Provides Safe and Secure Internet Use for Young People, it stipulates measures not only related to foods but also to improving the ability of youth to properly use the internet, improving the filtering function of software against unhealthy information for youth, and its diffusion of availability. The Act further provides measures to minimize access to harmful information by youth and encourages the implementation of these measures into action.  <Policies>  1) As for policies for children, a symposium on advertisements targeted to children was organized by the Consumer Commission, Cabinet Office. In the report, various discussions were cited on the ethics of advertisement as a whole, not limited to foods.  2) As an example of the activities made by private or non-governmental organizations, Save the Children Japan, a public interest incorporated association, playing a central role, reviewed advertisements and marketing methods of various items including foods, and made guidelines that would contribute to promote respect for children's rights. |
| **International best practice benchmark(s)**  CHILE: The regulatory norms define advertising targeted to children as websites directed to children or with an audience of greater than 20% children, and according to the design of the advertisement. Promotional strategies and incentives, such as cartoons, animations, and toys that could attract the attention of children are included in the ban. The regulation took effect 1 July 2016 and applies to all advertising media.  QUEBEC (CANADA): In Quebec, most citizens speak French and it is the only province in Canada, where children below 13 years old are protected under the Consumer Protection Act since 1980. In Québec, the Consumer Protection Act prohibits commercial advertising directed at children less than 13 years of age through all media. To determine whether or not an advertisement is directed at persons under thirteen years of age, account must be taken of the context of its presentation, and in particular of: a) the nature and intended purpose of the goods advertised; b) the manner of presenting such advertisement; and c) the time and place it is shown. Any stakeholder involved in a commercial process may be accused of not complying with the legislation in force. Per indictment, that person is liable to: a fine ranging from $600 to $15,000 (in the case of a natural person); a fine ranging from $2,000 to $100,000 (in the case of a legal person).  LONDON: Junk food adverts are to be banned from London’s tube and bus network by Sadiq Khan, the city’s mayor, in an attempt to curb an epidemic of childhood obesity. Ads that promote foods and drinks that are high in salt, fat and sugar will no longer be accepted on tubes, buses and overground trains that are part of the Transport for London (TfL) network – nor in stations. The ban is currently out to consultation. |
| **PROMO3**: Effective policies are implemented by the government to ensure that unhealthy foods are not commercially promoted to children in settings where children gather (e.g. preschools, schools, sport and cultural events) |
| <Laws/Acts>  No law/act has been formulated.  <Policies>  In the report on the symposium to explore advertisements to children sponsored by the Consumer Commission, Cabinet Office, the government shared views with the private sector, by providing opportunities to explore ethics on advertisements targeted at children. |
| **International best practice benchmark(s)**  CHILE: In June 2015, the Chilean authority approved the regulatory norms required for the law’s implementation (Diario Oficial No 41.193). The regulatory norms define limits for calories, saturated fat, sugar and sodium content considered “high” in foods and beverages. The law restricts advertising directed to children under the age of 14 of foods in the “high in” category on school grounds, including preschools, primary and secondary schools. Promotional strategies and incentives, such as cartoons, animations, and toys that could attract the attention of children are included in the ban. The law is scheduled to take effect 1 July 2016.  SPAIN: In 2011 the Spanish Parliament approved a Law on Nutrition and Food Safety (Ley 17/2011), which stated that kindergartens and schools should be free from all advertising. Criteria for the authorisation of food promotion campaigns, nutritional education and promotion of sports or physical activity campaigns were developed jointly by the Spanish Agency for Consumer Affairs, Food Safety and Nutrition (AECOSAN) and the Regional Health Authorities and implemented in July 2015. AECOSAN and the Spanish Regional Education and Health Administrations monitor the enforcement of the law.  URUGUAY: In September 2013, the government of Uruguay adopted Law No 19.140 “Alimentación saludable en los centros de enseñanza” (Healthy foods in schools). The law prohibits the advertising and marketing of foods and drinks that don’t meet the nutrition standards [referenced in Article 3 of the law, and outlined in school nutrition recommendations published by the Ministry of Health in 2014]. Advertising in all forms is prohibited, including posters, billboards, and use of logos/brands on school supplies, sponsorship, and distribution of prizes, free samples on school premises and the display and visibility of food. The implementation of the law started in 2015. |
| **4 FOOD PRICES**: Food pricing policies (e.g., taxes and subsidies) are aligned with health outcomes by helping to make the healthy eating choices the easier, cheaper choices |
| **PRICES1**: Taxes or levies on healthy foods are minimised to encourage healthy food choices where possible (e.g. low or no sales tax, excise, value-added or import duties on fruit and vegetables) |
| <Laws/Acts>  In order to assist low-income people who would be affected by the 10% consumption tax increase enacted as of October 2019, various remedies related to a reduced tax rate system are introduced, mainly in the Tax Reform Act.  <Policies>  In order to assist low-income people in conjunction with the 10% consumption tax increase enacted as of October 2019, a reduced tax rate system is applied to basic daily foods/beverages, including fruits and vegetables. |
| **International best practice benchmark(s)**  AUSTRALIA: Goods and services tax (GST) exemption exists for basic foods (including fresh fruits and vegetables).  TONGA: In 2013, as part of a broader package of fiscal measures, import duties were lowered from 20% to 5% for imported fresh, tinned or frozen fish in order to increase affordability and promote healthier diets.  FIJI: To promote fruit and vegetable consumption, Fiji has removed the excise duty on imported fruits, vegetables and legumes. It has also decreased the import tax for most varieties from the original 32% to 5% (exceptions: 32% remains on tomatoes, cucumbers, potatoes, squash, pumpkin and 15% remains on coconuts, pineapples, guavas, mangosteens) and removed it for garlic and onions. |
| **PRICES2**: Taxes or levies on unhealthy foods (e.g. sugar-sweetened beverages, foods high in nutrients of concern) are in place and increase the retail prices of these foods by at least 10% to discourage unhealthy food choices where possible, and these taxes are reinvested to improve population health |
| <Laws/Acts> and <Policies>  No law/act and policies been formulated that places taxes on unhealthy foods, nor have policies been formulated.  Supplementary comments:  By conclusion of the Comprehensive and Progressive Agreement for Trans-Pacific Partnership (CPTTP Agreement), taxes on most food items were eliminated among member countries. As a result, accessibility to healthy imported foods (including vegetables and fruits) was increased as well as sweets and favorite articles. |
| **International best practice benchmark(s)**  MEXICO: In December 2013, the Mexican legislature passed two new taxes as part of the national strategy for the prevention of overweight, obesity and diabetes. An excise duty of 1 peso ($0.80) per litre applies to sugary drinks. Sugary drinks are defined under the new law as all drinks with added sugar, excluding milks or yoghurts. This is expected to increase the price of sugary drinks by around 10%. An ad valorem excise duty of 8% applies to foods with high caloric density, defined as equal to or more than 275 calories per 100 grams.  HUNGARY: A "public health tax" adopted in 2012 is applied on the salt, sugar and caffeine content of various categories of ready-to-eat foods, including soft drinks (both sugar- and artificially-sweetened), energy drinks and pre-packaged sugar-sweetened products. The tax is applied at varying rates. Soft drinks, for example, are taxed at $0.24 per litre and other sweetened products at $0.47 per litre. The tax also applies to products high in salt, including salty snacks with >1g salt per 100g, condiments with >5g salt per 100g and flavourings >15g salt per 100g.  FRENCH POLYNESIA: Various food and beverage taxes have been in place since 2002 to discourage consumption and raise revenue e.g. domestic excise duty on sweetened drinks and beer; import tax on sweetened drinks, beer and confectionery; tax on ice cream. Between 2002 and 2006, tax revenue went to a preventive health fund; from 2006, 80% has been allocated to the general budget and earmarked for health. The tax is 40 CFP (around $0.44) per litre on domestically-produced sweet drinks, and 60 CFP (around $0.68) per litre on imported sweet drinks. |
| **PRICES3**: The intent of existing subsidies on foods, including infrastructure funding support (e.g. research and development, supporting markets or transport systems), is to favour healthy rather than unhealthy foods |
| <Laws/Acts>  No particular law/act has been found that supports the promotion of healthy foods by providing subsidies. On the other hand, the Health Promotion Act makes its best efforts for health promotion practitioners and related parties to: disseminate correct knowledge on health promotion through education and publicity; collect/arrange/analyze/provide information on health promotion; promote research; train personnel and improve their quality to deal with health promotion; and provide necessary technical assistance to practitioners of health promotion and other related people.  <Policies>  1) In the budget of fiscal year 2019 to the Ministry of Health, Labour and Welfare submitted an estimate of 3.1 billion yen to be utilized for “health promotion and prevention of diseases for the extension of healthy life expectancy”, including good eating habits.  2) The Health and Labor Sciences Research Grants provided by the Ministry of Health, Labour and Welfare, invite themes related to food and health from the public every year, in order to promote administrative measures in a scientific way and to improve technical standards on such issues as health care, welfare, hygiene, occupational safety and health.  3) In the 2018 reform on the review system of the Grants-in-Aid for Scientific Research-KAKENHI under the Japan Society for the Promotion of Science, “nutrition science” was added to a subcategory of sports science, physics and health science, where issues related to nutrition were combined.  4) In the private sector, mainly food-related enterprises invite applications from the public for a research grant for healthy foods. |
| **International best practice benchmark(s)**  SINGAPORE: The government, through the Health Promotion Board (HPB) increases the availability and use of healthier ingredients through the “Healthier Ingredient Scheme” (formerly part of the "Healthier Hawker" programme, launched in 2011), which provides in the first instance transitional support to oil manufacturers and importers to help them increase the sale of healthier oils to the food service industry. The Healthier Ingredient Subsidy Scheme offers a subsidy to suppliers stocking healthier items. Cooking oil is the first ingredient under the scheme, which subsidises oils with a saturated fat level of 35 per cent or lower. |
| **PRICES4**: The government ensures that food-related income support programs are for healthy foods |
| <Laws/Acts> and <Policies>  No law/act and policies been formulated which ensures public assistance (program for low-income individuals) that be utilized for purchasing healthy foods.  Supplementary comments:  1) The Education Assistance and Learning Support System provide school lunch fees to people who received public assistance.  2) A FY2017 status of implementing free school lunch provision was found as follows: out of 1,740 municipal governments, 76 (4.4% of total) provided free lunch both at primary and junior high schools; four (0.2%) provided lunch at only primary schools, and two (0.1%) provided lunch at only junior high schools. |
| **International best practice benchmark(s)**  UK: The British Healthy Start programme provides pregnant women and/or families with children under the age of four with weekly vouchers to spend on foods including milk, plain yoghurt, and fresh and frozen fruit and vegetables. Participants or their family must be receiving income support/jobseekers allowance or child tax credits. Pregnant women under the age of 18 can also apply. Full national implementation of the programme began in 2006  US: 1) In 2012, the USDA piloted a "Healthy Incentives Pilot" as part of the Supplemental Nutrition Assistance Program (SNAP, formerly "food stamps"). Participants received an incentive of 30 cents per US$ spent on targeted fruit and vegetables (transferred back onto their SNAP card). The Pilot included 7500 individuals. In New York City and Philadelphia, “Health Bucks” are distributed to farmers markets. When customers use income support (e.g. Food Stamps) to purchase food at farmers markets, they receive one Health Buck worth 2USD for each 5USD spent, which can then be sued to purchase fresh fruit and vegetable products at a farmers market. In Philadelphia, the programme has been expanded to other retail settings like supermarkets and corner stores.  2) In 2009, the U.S. Department of Agriculture's implemented revisions to the Special Supplemental Nutrition Program for Women, Infants, and Children (WIC) to improve the composition and quantities of WIC-provided foods from a health perspective. The revisions include: Increase the dollar amount for purchases of fruits and vegetables, expand whole-grain options, allow for yoghurt as a partial milk substitute, allow parents of older infants to buy fresh produce instead of jarred infant food and give states and local WIC agencies more flexibility in meeting the nutritional and cultural needs of WIC participants. |
| **5 FOOD PROVISION**:  The government ensures that there are healthy food service policies implemented in government-funded settings to ensure that food provision encourages healthy food choices, and the government actively encourages and supports private companies to implement similar policies |
| **PROV1**: The government ensures that there are clear, consistent policies (including nutrition standards) implemented in schools and early childhood education services for food service activities (canteens, food at events, fundraising, promotions, vending machines etc.) to provide and promote healthy food choices |
| <Laws/Acts>  1) In the School Lunch Program Act, desirable standards called Practice Standards for School Lunch are set, which state the nutrition intake required for pupils and students and other necessary issues to implement school lunches (excluding issues stipulated in Item 1 of the next Article).  2) The Basic Act on Shokuiku (Food and Nutrition Education) stipulates that nationals should take every opportunity and location, either at home, school, nursery, community or other, to involve themselves in various activities and deepen understanding of food and related issues.  <Policies>  1) Based on the School Lunch Program Act, the Practice Standards for School Lunch are set average recommended dietary allowance for each meal per pupil/student.  2) Based on the Basic Act on Shokuiku (Food and Nutrition Education), the Third Basic Plan for Promotion of Shokuiku, a five-year plan, is formulated to promote policies related to food and education in a comprehensive and systematic manner.  3) Staff/head of nursery schools, staff-in-charge of municipal governments and those engaged in the providing meals are recommended to make use of the Guidelines for food provision at nursery school in order to enrich meals.  4) In compliance with the Health Promotion Act, guidance and support are provided by public health center for Specification Lunch Facilities that provide lunch at schools, kindergartens, and nursery schools, so that proper nutrition management is maintained in those facilities. Among the guidance/support provided on activities related to health promotion in FY2017, nutrition guidance was found to be most frequently conducted, followed by exercise guidance. |
| **International best practice benchmark(s)**  CHILE: In 2012, the Chilean government approved a Law of Nutritional Composition of Food and Advertising (Ley 20, 606). In June 2015, the Chilean authority approved the regulatory norms required for the law’s implementation (Diario Oficial No 41.193). The regulatory norms define limits for calories, saturated fat, sugar and sodium content considered “high” in foods and beverages. The law prohibits the sale of foods in the “high in” category in schools. These were scheduled to take effect 1 July 2016.  FINLAND: In 2008, the National Nutrition Council approved nutrition recommendations for school meals. These include food and nutrient recommendations for salt, fibre, fat, starch, fat and salt maximums for meat and processed meat, and drinks. There are also criteria for snacks provided in schools. In 2017, the Finnish National Nutrition Council updated their nutrition recommendations for school meals replacing the recommendations from 2008. The updated recommendations are based on Health from food – Finnish nutrition guidelines (2014) and Eating Together – food recommendations for families with children (2016). No soft drinks, energy drinks or any other acidified beverages or beverages with added sugar are served at schools. |
| **PROV2**: The government ensures that there are clear, consistent policies in other public sector settings for food service activities (canteens, food at events, fundraising, promotions, vending machines, public procurement standards etc.) to provide and promote healthy food choices |
| * No law/act has been formulated, which regulates the kinds of foods provided at events, fund-raising, sales promotions and vending machines.  <Laws/Acts>  1) Based on the Health Promotion Act, Specification Lunch Facilities are defined to be those facilities that require nutrition management in providing meals constantly to specific and numerous people, as stipulated in the Ordinance of the Ministry of Health, Labour and Welfare.  2) According to the Regulations for Enforcement of the Health Promotion Act, those facilities stipulated by Article 20, Item 1 under the Ordinance of the Ministry of Health, Labour and Welfare, are assigned as Specification Lunch Facilities which offer more than 100 meals each time or more than 250 meals per day. It is recommended that guidance by nutritionists or registered nutritionists be given to those facilities.  <Policies>  1) The Ministry of Health, Labour and Welfare gave notice on Guidance and Support on Nutrition Management in Specific Food Service Facilities, and pointed out some issues regarding efficient and effective guidance/support for the administration of municipal governments, based on the current analysis of those facilities.  2) Policies are being implemented to make it easy to select healthy foods, as promoted by the Third Basic Plan for Promotion of Shokuiku and Health Japan 21 (the second term). |
| **International best practice benchmark(s)**  LATVIA: In 2012, the government set salt levels for all foods served in hospitals and long-term social care institutions. Levels may not exceed 1.25g of salt per 100g of food product; fish products may contain up to 1.5g of salt per 100g of product.  BERMUDA: In 2008, the Government Vending Machine Policy was implemented in government offices and facilities to ensure access to healthy snacks and beverages for staff. The policy requires that all food and beverages in vending machines on government premises meet specific criteria based on levels of total fat, saturated fat, trans fat, sodium and sugar. Criteria exclude nuts & 100% fruit juices.  NEW YORK (US): New York City’s Food Standards (enacted with Executive Order 122 of 2008) set nutritional standards for all food purchased or served by city agencies, which applies to prisons, hospitals and senior care centres. The Standards include: maximum and minimum levels of nutrients per serving; standards on specific food items (e.g. only no-fat or 1% milk); portion size requirements; the requirement that water be offered with food; a prohibition on the deep-frying of foods; and daily calorie and nutrient targets, including population-specific guidelines (e.g. children, seniors). |
| **PROV3**: The Government ensures that there are good support and training systems to help schools and other public sector organisations and their caterers meet the healthy food service policies and guidelines |
| <Laws/Acts>  1) As stipulated in the School Education Act, it is possible to rearrange part of school duties and assign a principal teacher to be in charge of children’s care or guidance/management of nutrition, if it is found to be necessary, depending on the situation of schools.  2) The Long-Term Care Insurance Act is designed for the needy to be an independent life, by the provision of Health and Medical Services and Welfare Services. This is aimed at improving health care and promoting welfare services for nationals.  <Policies>  1) Based on the School Education Act, nutrition teachers are expected to prepare a comprehensive plan on food guidance and to play a key role in the promotion of food and education. For this purpose, nutrition teachers at public schools are given training by municipal governments as part of the Nutrition Teacher System.  2) In order to promote children’s basic life habits such as proper exercise, a balanced diet, enough rest/sleep and other, the Ministry of Education, Culture, Sports, Science and Technology offers an award to municipal governments that organized excellent educational activities.  3) In order to promote meal delivery service that promote health support targeted at local aged people, guidelines on nutrition management for meal delivery service.  4) Visit instruction for individuals and training workshop on proper nutrition management, targeted for Specific Food Service Facilities, are provided by municipal governments where public health centers are located.  5) In some municipal governments, public health centers offer the visit instruction and training workshop on the proper nutrition management for Specific Food Service Facilities. |
| **International best practice benchmark(s)**  AUSTRALIA: The Healthy Eating Advisory Service supports settings such as childcare centres, schools, workplaces, health services, food outlets, parks and sporting centres to provide healthy foods and drinks to the public in line with Victorian Government policies and guidelines. The Healthy Eating Advisory Service is delivered by experienced nutritionists and dieticians at Nutrition Australia Victorian Division. The support includes training cooks, chefs, foods service and other key staff, discovering healthier recipes, food ideas and other helpful resources to provide healthier menus and products. |
| **PROV4**: The Government actively encourages and supports private companies to provide and promote healthy foods and meals in their workplaces |
| <Laws/Acts>  The Industrial Safety and Health Act makes its best efforts of the management to provide necessary information, secure health-education instructors and improve their quality and give other support at work places, in order to maintain and improve workers’ health properly and effectively.  <Policies>  1) The action for healthcare industry was formulated to promote the extension of healthy life expectancy and to create new industries utilizing prevention and health management services (such as exercise, nutrition, health care services, etc.), which are not covered by public health insurance. It is specifically encouraged to promote strategically Health and Productivity Management for workers in a management view point.  2) As examples of policies and assessment indicators by the population approach, the number of healthy menus in canteens, the number of viewers on food improvement information, employees’ satisfaction rates on support services, etc., are cited in guidebooks of Health and Productivity Management.  3) The Ministry of Health, Labour and Welfare broadly publicizes the Nutritional improvement campaigns, so that various industries and associations, as main actors, expand this movement nation-wide to meet the objectives set in the main activities in an effective way and to promote cooperative relations among the parties concerned, under the slogan of the Smart Life Project “Let’s Extend healthy life expectancy”.  4) Nutrition guidance is provided by public health centers at work places with Specific Food Service Facilities. |
| **International best practice benchmark(s)**  SINGAPORE: The National Workplace Health Promotion Programme, launched in Singapore in 2000, is run by the Health Promotion Board. Both private and public institutions are encouraged to improve the workplace environment by providing tools and grants. Grants are awarded to help companies start and sustain health promotion programmes. Tools include a sample Healthy Workplace Nutrition Policy, a sample Healthy Workplace Catering Policy, and a detailed Essential Guide to Workplace Health, setting out ways to transform the workplace into a health-supporting work environment.  VICTORIA (AUSTRALIA): ‘Healthy choices: healthy eating policy and catering guide for workplaces’ is a guideline for workplaces to support them in providing and promoting healthier foods options to their staff. The guideline is supported by the Healthy Eating Advisory Service that helps private sector settings to implement such policies. Menu assessments and cook/caterer training are available free of charge to some eligible workplaces. |
| **6 FOOD IN RETAIL**:  The government has the power to implement policies and programs to support the availability of healthy foods and limit the availability of unhealthy foods in communities (outlet density and locations) and in-store (product placement) |
| **RETAIL1**: Zoning laws and policies are robust enough and are being used, where needed, by local governments to place limits on the density or placement of quick serve restaurants or other outlets selling mainly unhealthy foods in communities |
| <Laws/Acts> and <Policies>  No zoning laws and policies have been formulated that would limit the construction of fast-food chains and the opening of unhealthy food outlets. |
| **International best practice benchmark(s)**  SOUTH KOREA: In 2010 the Special Act on Children’s Dietary Life Safety Management established the creation of ‘Green Food Zones’ around schools, banning the sale of foods (fast food and soda) deemed unhealthy by the Food and Drug Administration of Korea within 200 metres of schools. In 2016, Green Food Zones existed at over 10000 schools.  US: In Detroit, the zoning code prohibits the building of fast food restaurants within 500 ft. of all elementary, junior and senior high schools.  UK: Around 15 local authorities have developed “supplementary planning documents” on the development of hot food takeaways. The policies typically exclude hot food takeaways from a 400m zone around the target location (e.g. primary schools). For example, Barking and Dagenham’s Local Borough Council, London, adopted a policy in 2010 restricting the clustering of hot food takeaways and banning them entirely from 400m exclusion zones around schools. In 2009, the Local Borough Council of Waltham Forest, London developed a planning policy in 2009 restricting the development of hot food takeaways in local centres, and excluding them completely from areas within 10min walks from schools, parks or other youth centres. St Helens Council adopted a planning document in 2011 and Halton in 2012. |
| **RETAIL2**: Zoning laws and policies are robust enough and are being used, where needed, by local governments to encourage the availability of outlets selling fresh fruit and vegetables |
| <Laws/Acts> and <Policies>  No zoning laws and policies have been formulated that would promote the availability of outlets selling fresh fruits and vegetables.  Supplementary Comments:  <Laws/Acts>  The Health Promotion Act states that prefectural governments should establish a basic plan called Prefectural Health Promotion Plans in order to promote health among residents.  <Policies>  According to the Prefectural Health Promotion Plans, governments support access to outlets selling healthy foods, by making municipality-certified labeling available for cooked dishes and packed lunches that contain vegetables. |
| **International best practice benchmark(s)**  US: In February 2014 the US Congress formally established the Healthy Food Financing Initiative (following a three year pilot) which provides grants to states to provide financial and/or other types of assistance to attract healthier retail outlets to underserved areas. The pilot distributed over 140 million USD in grants to states to provide financial and other types of assistance to attract healthier retail outlets in underserved areas. To date, 23 US states have implemented financing initiatives. For example, the New Jersey Food Access Initiative provides affordable loans and grants for costs associated with building new supermarkets, expanding existing facilities, and purchasing and installing new equipment for supermarkets offering a full selection of unprepared, unprocessed, healthy foods in under-served areas; the initiative targets both for-profit and not-for-profit organisations and food cooperatives.  SCOTLAND: In 2004, a small group of suppliers and retailers in Scotland established a pilot project called Healthy Living Neighbourhood Shops to increase the availability of healthier food options throughout Scotland, in both deprived and affluent areas, where little or no option existed to buy. The programme received funding from the Scottish Executive and worked closely with the Scottish Grocers’ Federation, which represents convenience stores throughout Scotland. Through a number of different trials, the programme established clear criteria for increasing sales and also developed bespoke equipment/point of sale (POS) materials which were given to participating retailers free of charge. This has led to around 600 convenience stores across Scotland improving their range, quality and stock of fresh fruit and vegetables and other healthier eating products. |
| **RETAIL3**: The Government ensures existing support systems are in place to encourage food stores to promote the in-store availability of healthy foods and to limit the in-store availability of unhealthy foods |
| <Laws/Acts> and <Policies>  No laws/acts and policies have been formulated that would encourage placing healthy foods, and discourage placing unhealthy foods, in food service outlets. |
| **International best practice benchmark(s)**  US: The Special Supplemental Nutrition Program for Women, Infants, and Children (WIC) requires WIC authorised stores to stock certain healthier products (e.g. wholegrain bread). |
| **RETAIL4**: The government ensures existing support systems are in place to encourage food service outlets to increase the promotion and availability of healthy foods and to decrease the promotion and availability of unhealthy foods |
| <Laws/Acts> and <Policies>  No laws/acts and policies have been formulated that would encourage food service outlets to increase the promotion and availability of healthy foods and to decrease those of unhealthy foods. |
| **International best practice benchmark(s)**  US: In December 2011, San Francisco implemented the Health Food Incentives Ordinance which bans restaurants, including takeaway restaurants, to give away toys and other free incentive items with children’s meals unless the meals meet nutritional standards as set out in the Ordinance: meals must not contain more than 600 calories, 640mg sodium, 0.5g trans fat, 35% total calories from fat and 10% calories from saturated fat and include a min amount of fruits and vegetables, while single food items and beverages must have <35% total calories from fat and <10% of calories from added caloric sweeteners. Incentives are defined as physical and digital items that appeal to children and teenagers as well as coupons, vouchers or similar which allow access to these items. In 2010 Santa Clara county, California banned restaurants from providing toys or other incentives with m menu items high in calories, sodium, fast or sugars. The law (Ordinance No NS300-820) sets nutrition standards prohibiting restaurants from linking toys or other incentives with single food items or meals with excessive calories (more than 200 for single food items and more than 485 calories for meals), excessive sodium (more than 480mg for single food item and more than 600mg for a meal), excessive fat (more than 35% for total fat), excessive saturated fat (>10%) and sugar ( more than 10% total calories from caloric sweeteners) or more than 0.5g of trans fats. It also applies to drinks with excessive calories (more than 120 calories) and fat (more than 35% from fat) and excessive sugars (more than 10% from caloric sweeteners) added non-nutritive sweeteners or caffeine.  FRANCE: Since January 2017 France has banned unlimited offers of sweetened beverages for free or at a fixed price in public restaurants and other facilities accommodating or receiving children under the age of 18. Sweetened beverages are defined as any drink sweetened with sugar or artificial (caloric and non-caloric ) sweeteners , including flavoured carbonated and still beverages, fruit syrups, sport and energy drinks, fruit and vegetable nectars, fruit- and vegetable-based drinks, as well as water- milk- or cereal-based beverages. |
| **7 FOOD TRADE AND INVESTMENT**:  The government ensures that trade and investment agreements protect food sovereignty, favour healthy food environments, are linked with domestic health and agricultural policies in ways that are consistent with health objectives, and do not promote unhealthy food environments |
| **TRADE1**: The Government undertakes risk impact assessments before and during the negotiation of trade and investment agreements, to identify, evaluate and minimize the direct and indirect negative impacts of such agreements on population nutrition and health |
| <Laws/Acts>  In order to prevent risks arising from the hygiene of foods/drinks and to protect public health, the Food Sanitation Act stipulates that those who wish to import foods, additives, equipment or packages, should report to the Minister of Health, Labour and Welfare at each occurrence of import, in compliance with the Ordinance of the Ministry of Health, Labour and Welfare.  <Policies>  The Ministry of Health, Labour and Welfare and quarantine stations enhance inspection systems at import, such as adding checkout items, maintaining inspection equipment, and strengthening monitoring and instruction including inspection orders. By addressing the security of imported foods, the Ministry ensures the safety of food and protects health of citizens. |
| **International best practice benchmark(s)**  US/EU: It is mandatory in the US and countries of the EU to undertake Environmental Impact Assessments for all new trade agreements. These assessments sometimes incorporate Health Impact Assessments. |
| **TRADE2**: The government adopts measures to manage investment and protect their regulatory capacity with respect to public health nutrition |
| <Laws/Acts> and <Policies>  *No laws/acts and policies have been formulated to protect regulatory capacity for public health and nutrition. Some issues on investment management are described as below.    <Laws/Acts>  1) Based on the Health Promotion Act, subsidies are provided to participating prefectures and municipalities to make up part of the expenses spent on promotional activities for health of citizens  2) The Industrial Safety and Health Act encourages management to provide necessary support for workers at workplaces, such as obtaining instructors on health education to improve their quality. Based on this Act, Health and Productivity Management indicated that workers’ health management should be considered from a management perspective and implemented in a strategic manner. Health and Productivity Management is regarded as part of measures to deal with the extension of healthy life expectancy of nationals and is incorporated in Japan’s reconstruction strategy and future investment strategy.  3) Nutrition management required by meal delivery service that are engaged in promoting health support to local aged people, is stipulated in the Guidelines on Nutrition Management for Food Delivery Service.  <Policies>  1) Based on the report from the Investigative Commission on Healthy Foods to Support the Healthy and Long-lived Society, a symbol mark to promote “healthy foods” was adopted. This mark would help consumers to see at a glance that those food products on the market (cooked foods) comply with the healthy food standards. The mark would also make them easily accessible and they could be combined to eat with other dishes as necessary.  2) The Nutritional improvement campaigns has been conducted annually since 2016. In 2018, this campaign was conducted nationwide across municipal governments for one month, with the basic theme “eat nicely with a good balance”, in order to increase the intake of vegetables, decrease that of salt, and lead to a habitual intake of milk and dairy products. |
| **International best practice benchmark(s)**  MANY COUNTRIES: Sanitary and phytosanitary (SPS) clauses in World Trade Organization (WTO) agreements. However, this usually does not apply to public health nutrition.  GHANA: Ghana has set standards to limit the level of fats in beef, pork, mutton and poultry in response to rising imports of low quality meat following liberalization of trade. The relevant standards establish maximum percentage fat content for de-boned carcasses/cuts for beef (<25%), pork (<25%) and mutton (<25% or <30% where back fat is not removed), and maximum percentage fat content for dressed poultry and/or poultry parts (<15%). |

**Infrastructure-support component**

| **8 LEADERSHIP**:  The political leadership ensures that there is strong support for the vision, planning, communication, implementation and evaluation of policies and actions to create healthy food environments, improve population nutrition, and reduce diet-related inequalities |
| --- |
| **LEAD1**: There is strong, visible, political support (at the Head of Government / Cabinet level) for improving food environments, population nutrition, diet-related NCDs and their related inequalities |
| <Laws/Acts>  The Health and Medical Strategy Advancement Act states that the nation should formulate polices on advanced research/development and new technology creation in a comprehensive and constructed way, and be responsible for its implementation. The purpose is to contribute to the growth of the Japanese economy, while contributing to the improvement of overseas medical quality, by creating new industrial activities aimed at a healthy and long-lived society, promoting the same in foreign countries and activating such programs.  <Policies>  1) Based on the Health and Medical Strategy Advancement Act, the government raised three action plans for growth strategy, one of which is the Strategic Market Creation Plan. In this plan, the extension of healthy life expectancy among nationals is considered as one of the themes, and hence, the government has adopted the following policies to produce an ideal society towards 2030.  1. To lead a healthy life until old age, by providing effective preventive services and health management.  2. To receive the world’s most advanced medical services, by galvanizing medical related industries.  3. To return to society soon after sickness or injuries, by having access to quality medical and care services.  2) Viewing a global trend to improve nutrition, the government formulates the following strategies to implement and enforce nutrition improvement.  1. Towards implementation of nutrition improvement globally, to examine methods of formulating nutrition improvement by individual companies and government support policies.  2. To contribute to the improvement of nutrition by liaising with polices of respective ministries.  3. To disseminate knowledge and experience on nutrition policies, and to contribute to a global society, by establishing a working team under the task force, for the international development of medical care.  3) The Headquarters for Healthcare Policy in the Cabinet Secretariat established Promotion Council for Healthcare Policy to support nutrition improvement projects. The working group was set up under the international medical development taskforce, that is, Headquarters for Healthcare Policy, the Ministry of Foreign Affairs, the Ministry of Education, Culture, Sports, Science and Technology, the Ministry of Health, Labour and Welfare, the Ministry of Agriculture, Forestry and Fisheries, the Ministry of Economy, Trade and Industry and other related ministries. The working group is examining current situations and issues related to nutrition and is preparing for the 2020 Summit Meeting on Nutrition, which is to promote global actions to deal with nutrition issues. |
| **International best practice benchmark(s)**  NEW YORK (US): As Mayor of New York City, Michael Bloomberg prioritised food policy and introduced a number of ground breaking policy initiatives including ‘Health Bucks’, a restriction on trans fats, establishment of an obesity taskforce, a portion size restriction on sugar-sweetened beverages, public awareness campaigns, etc. He showed strong and consistent leadership and a commitment to innovative approaches and cross-sectoral collaboration.  BRAZIL: The Minister of Health showed leadership in developing new dietary guidelines that are drastically different from the majority of dietary guidelines created by any nation to date, and align with some of the most commonly cited recommendations for healthy eating.  CARICOM COUNTRIES: Active NCD commissions exist in six of the 20 CARICOM member states (Bahamas, Barbados, Bermuda, British Virgin Islands, Dominica, Grenada) which are all housed in their Ministries of Health, with members recommended by the Minister of Health and appointed by the Cabinet of Government for a fixed duration; all include government agencies and to a varying degree, civil society and the private sector. |
| **LEAD2**: Clear population intake targets have been established by the government for the nutrients of concern to meet WHO and national recommended dietary intake levels |
| <Laws/Acts>  The Health Promotion Act stipulates that in order to promote public health in a comprehensive manner, the Minister of Health, Labour and Welfare should present 1) Basic goals for implementation of National Health Promotion and 2) Items relating to targets in public health promotion.  <Policies>  1) In order to present some basic issues for health promotion in a comprehensive manner, Health Japan 21 (the second term) was incorporated in the Basic Direction for Comprehensive Implementation of National Health Promotion.  2) Health Japan 21 (the second term) includes the Section of Nutrition and dietary habits “Targets for improvement of everyday habits and social environment relating to nutrition and dietary habits, physical activity and exercise, rest, alcohol, smoking, and dental and oral health”. There are targets to decrease the salt and fat intake and to increase registered food industries and shops that try to contain salt and fat in foods. |
| **International best practice benchmark(s)**  BRAZIL: The "Strategic Action Plan for Confronting NCDs in Brazil, 2011-2022 specifies a target of increasing adequate consumption of fruits and vegetables, from 18.2% to 24.3 % between 2010 and 2022 and reduction of the average salt intake of 12 g to 5 g, between 2010 and 2022.  SOUTH AFRICA: The South African plan for the prevention and control of non-communicable diseases includes a target on reducing mean population intake of salt to <5 grams per day by 2020.  UK: In July 2015, the government adopted as official dietary advice the recommendation of the Advisory Committee on Nutrition that sugar should make up no more than 5% of daily calorie intake (30g or 7 cubes of sugar per day). Current sugar intake makes up 12 to 15% of energy. An evidence review by Public Health England outlines a number of strategies and interventions. In August 2016 the Government published ‘Childhood obesity: A plan for action’. This included a commitment for Public Health England (PHE) to oversee a sugar reduction programme. This challenges all sectors of the food industry to reduce by 20% by 2020 the level of sugar in the categories that contribute most to the intakes of children up to 18 years. |
| **LEAD3**: Clear, interpretive, evidence-informed food-based dietary guidelines have been established and implemented |
| <Laws/Acts>  In the Basic Act on Shokuiku (Food and Nutrition Education), it is stipulated to nurture a sound judgement in selecting suitable foods for a healthy diet.  <Policies>  1) The Dietary guidelines recommends the following balanced diet in the level of food and cooked meal; Eat well-balanced meals with staple food, as well as main and side dishes; Eat enough grains such as rice and other cereals; Combine vegetables, fruits, milk products, beans and fish in your diet; and Avoid too much salt. Attention should be paid to the quality and quantity of fat ingested.  2) Based on the Dietary guidelines, the Japanese Food Guide recommends combinations of dishes and servings with illustrations, in order to give guidance on what, and how much, to take per day, in a simple way. |
| **International best practice benchmark(s)**  BRAZIL: The national dietary guidelines of Brazil address healthy eating from a cultural, ethical and environmental perspective, rather than based on number of servings per food group. The main recommendations are: ‘Make natural or minimally processed foods the basis of your diet’; ‘use oils, fats, salt, and sugar in small amounts for seasoning and cooking foods’; ‘use processed foods in small amounts’; ‘avoid ultra-processed foods’. They also provide advice on planning, shopping and sharing meals, as well as warning people to be wary of food marketing and advertising. |
| **LEAD4**: There is a comprehensive, transparent, up-to-date implementation plan (including priority policy and program strategies, social marketing for public awareness and threat of legislation for voluntary approaches) linked to national needs and priorities, to improve food environments, reduce the intake of the nutrients of concern to meet WHO and national recommended dietary intake levels, and reduce diet-related NCDs |
| <Laws/Acts> and <Policies>  *No laws/acts and policies have been formulated as to legal restrictions against unfavorable activities on health.  <Laws/Acts>  The Health Promotion Act states that the Minister of Health, Labour and Welfare demonstrates 1) Basic goals for implementation of National Health Promotion and 2) Items relating to targets in public health promotion, in order to promote the health promotion in a comprehensive manner.  <Policies>  The Health and Science Council (the Promotion Committee of Health Japan 21 [the second term]) publicized an interim report. Based on the findings, it presented some issues to be solved and the latest implementation plan to increase the intake of vegetables and fruits and decrease that of salt and fat, which are designated as one of the nutrients of concern. |
| **International best practice benchmark(s)**  EU: The European Food and Nutrition Action Plan 2015-20 outlines clear strategic goals, guiding principles, objectives, priorities and tools. The Plan aligns with the WHO Global Action Plan and under ‘Objective 1 – Create healthy food and drink environments’ there are clear policy and program actions identified. |
| **LEAD5**: Government priorities have been established to reduce inequalities or protect vulnerable populations in relation to diet, nutrition , obesity and NCDs |
| <Laws/Acts>  The Health Promotion Act stipulates that the Minister of Health, Labour and Welfare is to formulate the Basic Direction for Comprehensive Implementation of National Health Promotion.  <Policies>  1) In the Basic Direction for Comprehensive Implementation of National Health Promotion presented by the Ministry of Health, Labour and Welfare, it states the Basic goals for implementation of National Health Promotion to reduce health disparities (i.e., gap in health status between the groups, created by difference in community or socioeconomic status), through development of good social environment which supports health life at every life stage.  2) The government promotes measures to improve diet and habituate exercise, focused on primary prevention (i.e., preventing noncommunicable diseases and promoting health by improving life styles), and to prevent cancer, cardiovascular [disease](https://eow.alc.co.jp/search?q=disease&ref=awlj)s, diabetics and chronic obstructive pulmonary disease. |
| **International best practice benchmark(s)**  NEW ZEALAND: The Ministry of Health reports the estimates derived from health surveys and nutrition surveys by four subpopulation groups including age group, gender, ethnic group and an area level deprivation index. Similarly, estimates derived from other data types (e.g. mortality) are presented by these subpopulation groups. The contracts between MoH and NGOs or other institutions include a section on Maori Health and state: “An overarching aim of the health and disability sector is the improvement of Maori health outcomes and the reduction of Maori health inequalities. You must comply with any: a) Maori specific service requirements, b) Maori specific quality requirements and c) Maori specific monitoring requirements”. In addition, the provider quality specifications for public health services include specific requirements for Maori:” C1 Services meet needs of Maori, C2 Maori participation at all levels of strategic and service planning, development and implementation within organisation at governance, management and service delivery levels, C3: support for Maori accessing services”.  AUSTRALIA: The National Indigenous Reform Agreement (Closing the Gap) is an agreement between the Commonwealth of Australia and the States and Territories. The objective of this agreement is to work together with Indigenous Australians to Close the Gap in Indigenous disadvantage. The targets agreed to by COAG relate to health or social determinants of health. For the target ‘Closing the life expectancy gap within a generation (by 2031)’, one of the performance indicators is the prevalence of overweight and obesity. |
| **9 GOVERNANCE**:  Governments have structures in place to ensure transparency and accountability, and encourage broad community participation and inclusion when formulating and implementing policies and actions to create healthy food environments, improve population nutrition, and reduce diet-related inequalities |
| **GOVER1**: There are robust procedures to restrict commercial influences on the development of policies related to food environments where they have conflicts of interest with improving population nutrition |
| <Laws/Acts>  Norms of conduct to be followed by government employees in case of conflicts of interest are regulated in detail in the Constitution of Japan, National Public Service Act, National Public Service Ethics Act and National Public Service Ethics Code.  <Policies>  In the official site of the National Personnel Authority, service disciplines and rules that a national government official should complied with. |
| **International best practice benchmark(s)**  US: Mandatory and publicly accessible lobby registers exist at the federal level, as well as in nearly every state. Financial information must be disclosed, and the register is enforced through significant sanctions. A number of pieces of legislation uphold compliance with the register including Lobbying Disclosure Act of 1995 and the Honest Leadership and Open Government Act 2007.  NEW ZEALAND: The State Services Commission has published Best Practice Guidelines for Departments Responsible for Regulatory Processes with Significant Commercial Implications. They cover the development and operation of a regulatory process and include specific references to principles around stakeholder relationship management.  AUSTRALIA: The Australian Public Service Commission’s Values and Code of Conduct includes a number of relevant sections such as the Conflict of Interest, Working with the Private Sector and other Stakeholders and the Lobbying Code of Conduct. |
| **GOVER2**: Policies and procedures are implemented for using evidence in the development of food policies |
| <Laws/Acts>  1) The National Government Organization Act and Act for Establishment of the Cabinet Office made it possible to set up a council system, in order to conduct study and deliberation, administrative appeal, and other consensual decision making involving academic experts, in case important issues are explored in formulating government policies.  2 In the Act Establishing the Ministry of Health, Labour and Welfare and Ordinance of the Health and Science Council, it is stipulated to select expert committee members from academic circles.  <Policies>  1) The Cabinet Legislation Bureau shows a process of formulating legislations, citing six phases from drafting to announcement, which include deliberations in working groups (consultation) composed of academic members.  2) Issues related to Health Japan 21 (the second term) are explored and assessed by Promotion Expert Committee of Health Japan 21 (the second term), that was adopted at the Health Promotion and Nutrition Section, Health Sciences Council.  3) There are instances of administrative assessment by the Ministry of Internal Affairs and Communications, and of promotion of an evidence-based policy formulation by the Headquarters for the Promotion of Administrative reform Cabinet Secretariat. |
| **International best practice benchmark(s)**  AUSTRALIA: The National Health and Medical Research Council Act 1992 (NHMRC Act) requires NHMRC to develop evidence-based guidelines. These national guidelines are developed by teams of specialists following a rigorous nine-step development process. |
| **GOVER3**: Policies and procedures are implemented for ensuring transparency in the development of food policies |
| <Laws/Acts>  There is no law/act to ensure transparency in formulating policies; on the other hand, procedures to formulate policies are the same as those stated in GOVESR2.  <Policies>  1) The Headquarters for the Promotion of Administrative reform Cabinet Secretariat occasionally reviews procedures on how to use objective facts and scientific data with regard to food-related policies, in order to enforce policies and administration  2) To ensure equality and improve transparency, a public comment system is adopted. |
| **International best practice benchmark(s)**  AUSTRALIA/NEW ZEALAND: Food Standards Australia New Zealand (FSANZ) is required by the Food Standards Australia New Zealand Act 1991 to engage stakeholders in the development of new standards. This process is open to everyone in the community including consumers, public health professionals, and industry and government representatives. FSANZ has developed a Stakeholder Engagement Strategy 2013-16 that outlines the scope and processes for engagement. Under the Stakeholder Engagement Priorities 2013-16, it outlined “maintain our open and transparent approach” as one of the first priorities. |
| **GOVER4**: The government ensures access to comprehensive nutrition information and key documents (e.g. budget documents, annual performance reviews and health indicators) for the public |
| <Laws/Acts>  Based on the citizens’ rights to seek for information disclosure and by the Act on Access to Information Held by Administrative Organs, the government discloses information under its possession and promotes to fulfill its accountability of explaining such activities.    <Policies>  1) The Act on Access to Information Held by Administrative Organs stipulates that nationals have the right to seek for disclosure of administrative documents, in order to promote fair and democratic administration under understanding and criticism from nationals.  2) In Basic Principles on Open Data, the Ministry of Health, Labour and Welfare publicizes an annual budget and a summary of settlement of expenses/taxes, a White Paper on Health, Labour and Welfare, and the National Health and Nutrition Survey.  3) Results of government generated statistics, such as the National Health and Nutrition Survey, are publicized on e-Stat. |
| **International best practice benchmark(s)**  AUSTRALIA/NEW ZEALAND: The Freedom of Information Act provides a legally enforceable right of the public to access documents of government departments and most agencies. |
| **10 MONITORING AND INTELLIGENCE**:  The government’s monitoring and intelligence systems (surveillance, evaluation, research and reporting) are comprehensive and regular enough to assess the status of food environments, population nutrition and diet-related NCDs and their inequalities, and to measure progress on achieving the goals of nutrition and health plans |
| **MONIT1**: Monitoring systems, implemented by the government, are in place to regularly monitor food environments (especially for food composition for nutrients of concern, food promotion to children, and nutritional quality of food in schools and other public sector settings), against codes/guidelines/standards/targets |
| <Laws/Acts>  Based on the Practice Standards for School Lunch and the standards for school lunch health administration stated in the School Lunch Program Act, a school lunch menu is designed. In the standards for school lunch health administration, hygiene inspection is required to be conducted on a regular and daily basis. On the other hand, Practice Standards for School Lunch is not required in the standards for school lunch implementation.  <Policies>  1) Procedures to design a school-lunch menu vary depending on municipalities. In general, they are as follows: 1) determine a menu (meal plan), 2) provide meals, 3) evaluate provided meals and 4) improve meal contents based on evaluation.  2) There is no regular monitoring on nutrition components in nutrients of concern. On the other hand, based on the school lunch intake standards, regular monitoring is conducted on nutrition quantity per meal for each pupil/student. |
| **International best practice benchmark(s)**  MANY COUNTRIES: Many countries do have food composition databases available. For example, the New Zealand Institute for Plant & Food Research Limited and the Ministry of Health jointly own the New Zealand Food Composition Database (NZFCD) which is a comprehensive collection of nutrient data in New Zealand containing nutrient information on more than 2600 foods.  NEW ZEALAND: A national School and Early Childhood Education (ECE) Services Food and Nutrition Environment Survey was organised in all Schools and ECES across New Zealand in 2007 and 2009 by the Ministry of Health to measure the food environments in schools and ECEs in New Zealand.  UK: in October 2005, the School Food Trust (‘the Trust’; now called the Children’s Food Trust) was established to provide independent support and advice to schools, caterers, manufacturers and others on improving the standard of school meals. They perform annual surveys, including the latest information on how many children are having school meals in England, how much they cost and how they’re being provided. |
| **MONIT2**: There is regular monitoring of adult and childhood nutrition status and population intakes against specified intake targets or recommended daily intake levels |
| <Laws/Acts>  The Health Promotion Act stipulates that the National Health and Nutrition Survey should be conducted, in order to clarify nutrition intakes and a lifestyle, which would be utilized as basic information for health promotion.  <Policies>  In the National Health and Nutrition Survey, the Ministry of Health, Labour and Welfare conducts an annual monitoring on health and nutrition among nationals, targeted at children to adults, to find out respective nutrition intakes and lifestyles. The purpose is to obtain basic information to enforce integrated promotional measures for public health. |
| **International best practice benchmark(s)**  US: The National Health and Nutrition Examination Survey (NHANES) is a program of studies designed to assess the health and nutritional status of adults and children in the United States. The survey is unique in that it combines interviews and physical examinations. The NHANES program began in the early 1960s and has been conducted as a series of surveys focusing on different population groups or health topics. In 1999, the survey became a continuous program that has a changing focus on a variety of health and nutrition measurements to meet emerging needs. The survey examines a nationally representative sample of about 5,000 persons each year. These persons are located in counties across the country, 15 of which are visited each year. |
| **MONIT3**: There is regular monitoring of adult and childhood overweight and obesity prevalence using anthropometric measurements |
| <Laws/Acts>  1) In the School Health and Safety Act, it is stipulated to conduct an annual health checkup for students and teachers, as is stated in the School Education Act.  2) In the Industrial Safety and Health Act, it is stipulated that the management should arrange medical examination by doctors for its employees.  <Policies>  1) In compliance with the School Education Act and the School Health and Safety Act, educational institutions conduct regular health checkups annually to maintain and promote the health of infants, children, pupils, students and school staff.  2) Adults are examined to determine if they are overweight or are obese. In recent years, health guidance, which is focused on visceral fat, is also conducted as necessary, by measuring abdominal circumference, blood pressure, fat, blood glucose, etc., and by checking the questionnaire (regarding treatment history, smoking and other lifestyle habits). |
| **International best practice benchmark(s)**  UK: England’s National Child Measurement Programme was established in 2006 and aims to measure all children in England in the first (4-5 years) and last years (10-11 years) of primary school. In 2011-2012, 565,662 children at reception and 491,118 children 10-11 years were measured. |
| **MONIT4**: There is regular monitoring of the prevalence of NCD risk factors and occurrence rates (e.g. prevalence, incidence, mortality) for the main diet-related NCDs |
| <Laws/Acts>  1) In the Regulation of patient survey, the investigation is conducted to clarify actual conditions of patients who use medical facilities.  2) In the Ordinance of Vital Statistics Survey, the Minister of Health, Labour and Welfare conducts demographic surveys based on reports of birth, death, stillbirth, marriage and divorce submitted by prefectures’ governors.  3) The Health Promotion Act stipulates that the state and local governments should try to find out background factors and conditions that could lead to non-communicable diseases.  4) The Act on the Promotion of Cancer Registries states that the government should grasp and analyze situations of cancer suffering, treatments and outcomes, and promote research on cancer-related issues.  <Policies>  1) In compliance with the Regulation of patient survey, the Patient survey (fundamental statistics) is conducted every year on categories of domestic hospitalization/outpatient and kinds of treatment, etc.  2) By the Ordinance of Vital Statistics Survey, household data on birth, death, stillbirth (and marriage and divorce) submitted in accordance with the Family Register Act and the Provisions on the notification of stillbirth, are collected monthly and publicized annually in early September of the next year. |
| **International best practice benchmark(s)**  OECD COUNTRIES: Most OECD countries have regular and robust prevalence, incidence and mortality data for the main diet-related NCDs and NCD risk factors. |
| **MONIT5**: There is sufficient evaluation of major programs and policies to assess effectiveness and contribution to achieving the goals of the nutrition and health plans |
| <Laws/Acts>  The Health Promotion Act stipulates that the Ministry of Health, Labour and Welfare should show Basic goals for implementation of National Health Promotion and Items relating to targets in public health promotion. These targets are assessed in accordance with the following policies.  <Policies>  1) The Ministry of Health, Labour and Welfare implements their policies in the management cycle, incorporating major factors of plan, do, check and action.  2) Policy assessment is classified according to the following categories: assessment of output, result of monitoring, integrated assessment, project assessment (before/after), public project assessment (before/after), and research and development assessment (before/after).  3) The Health Japan 21 (the second term) was designated for the duration of FY2013 through FY2022 campaign period, and an interim assessment was conducted in FY 2018 on all the targets, which was five years after implementation. |
| **International best practice benchmark(s)**  US: The National Institutes for Health (NIH) provide funding for rapid assessments of natural experiments. The funding establishes an accelerated review/award process to support time-sensitive research to evaluate a new policy or program expected to influence obesity related behaviours (e.g., dietary intake, physical activity, or sedentary behaviour) and/or weight outcomes in an effort to prevent or reduce obesity. |
| **MONIT6**: Progress towards reducing health inequalities or health impacts in vulnerable populations and societal and economic determinants of health are regularly monitored |
| <Laws/Acts>  1) According to the Comprehensive Survey of Living Conditions, basic surveys on lives of people are conducted to: investigate basic issues on lives such as health, medicine, welfare, pension, income; obtain necessary basic information to design and formulate policies: and establish a master sample to extract survey objects for various surveys.  2) By the Health Promotion Act, the state and local governments should make best efforts to grasp conditions that would lead to non-communicable diseases.  <Policies>  1) Based on the Comprehensive Survey of Living Conditions, basic surveys on national life are conducted annually on such basic issues as health, medicine, welfare, pension, income, etc.  2) Based on the Health Promotion Act, the National Health and Nutrition Survey is conducted to monitor items, such as physical condition, intake of nutrition, and Social Determinants of Health to reduce health disparities among nationals.  3) Utilizing the results from the Comprehensive Survey of Living Conditions and the National Health and Nutrition Survey, Health Japan 21 (the second term) conducted an interim assessment on all targets in FY2018, after five years from FY2013 when the program was initiated. Assessment was also made on one of the basic targets, the extension of healthy life expectancy and reduction of health disparity. |
| **International best practice benchmark(s)**  NEW ZEALAND: All annual Ministry of Health Surveys report estimates by subpopulations in particular by ethnicity (including Maori and Pacific peoples), by age, by gender, and by New Zealand area deprivation. |
| **11 FUNDING AND RESOURCES**:  Sufficient funding is invested in ‘Population Nutrition Promotion’(estimated from the investments in population promotion of healthy eating and healthy food environments for the prevention of obesity and diet-related NCDs, excluding all one-on-one promotion (primary care, antenatal services, maternal and child nursing services etc.), food safety, micronutrient deficiencies (e.g. folate fortification) and undernutrition) to create healthy food environments, improved population nutrition, reductions in obesity, diet-related NCDs and their related inequalities |
| **FUND1**: The ‘Population Nutrition Promotion’ budget, as a proportion of total health spending and/or in relation to the diet-related NCD burden is sufficient to reduce diet-related NCDs |
| <Laws/Acts>  The Health Promotion Act stipulates that the government is able to subsidize part of expenses within the budget ceiling for those projects that are conducted for health promotion based on Prefectural Health Promotion Plans and Municipal Health Promotion Plans.  <Policies>  In the budget of fiscal year 2019 to the Ministry of Health, Labour and Welfare, 3.1 billion yen budgets were allocated to the Extension of Healthy Life Expectancy for Preventing Disease and Health Promotion (equivalent to 17.5% of 17.7 billion yen for the Promotion of measures against extension of healthy life expectancy, passive smoking, hepatitis and intractable/rare disease) and 5.6 billion yen to the Cancer Genomic Medicine Promotion (equivalent to 31.6% of the same budget proposal). |
| **International best practice benchmark(s)**  NEW ZEALAND: The total funding for population nutrition was estimated at about $67 million or 0.6% of the health budget during 2008/09 Healthy Eating Healthy Action period. Dietary risk factors account for 11.4% of health loss in New Zealand.  THAILAND: According to the most recent report on health expenditure in 2012 the government greatly increased budget spent on policies and actions related to nutrition (excluding food, hygiene and drinking water control). Total expenditure on health related to nutrition specifically from local governments was 29,434.5 million Baht (about 840 million USD) (7.57% of total health expenditure from public funding agencies), which was ten times over the budget spending on nutrition in 2011. Dietary risk factors account for about 10% of health loss in Thailand. |
| **FUND2**: Government funded research is targeted for improving food environments, reducing obesity, NCDs and their related inequalities |
| <Laws/Acts>  1) The Health Promotion Act states the promotion of the research and provision by data collection, arrangement and analysis related to the health promotion.  <Policies>  1) The Health and Labour Sciences Research administered by the Ministry of Health, Labour and Welfare aims to ensure the scientific promotion of administration policies and to improve technical standards on Health and Medical Services, Welfare, Environmental Health, Industrial Safety and Health. The said research is divided into three areas, that is, policy and governance, measures against diseases and disability and integrated research for health and safety. Those areas include the improvement of food environment, decrease of obesity, and reduction of non-communicable diseases and other related issues aimed at the research that approaches against the reductions in health inequalities.  2) The National Research and Development Agency, Japan Agency for Medical Research and Development (AMED), promotes research and development, with a focus on nine integrated projects, including the creation of medical products, regenerative medicine, cancer, etc., based on the Research and Development Project for Medicine. These projects include researches on non-communicable diseases and related issues, aimed at the reduction in health inequalities. |
| **International best practice benchmark(s)**  AUSTRALIA: The National Health and Medical Research Council (NHMRC) Act requires the CEO to identify major national health issues likely to arise. The National Health Priority Areas (NHPAs) articulate priorities for research and investment and have been designated by Australian governments as key targets because of their contribution to the burden of disease in Australia. For the 2015-16 Corporate Plan, obesity, diabetes and cardiovascular health are three of these NHPAs.  THAILAND: The National Research Council funded more research projects on obesity and diet-related chronic diseases (such as diabetes, cardiovascular diseases and hypertension) in 2014, accountable for almost six times over the research funding in 2013 (from 6,875,028 Baht in 2013 to 37,872,416 baht in 2014). |
| **FUND3**: There is a statutory health promotion agency in place that includes an objective to improve population nutrition, with a secure funding stream |
| <Laws/Acts>  The Health Promotion Act states that practitioners of health promotion should make best efforts to implement projects that would enhance health education, health consultation and other necessary projects.  <Policies>  1) The National Institutes of Biomedical Innovation, Health and Nutrition, National Institute of Health and Nutrition, plays a role as a public institution to improve and promote public health, by conducting surveys and research on health maintenance and promotion as well as nutrition and dietary habit.  2) The National Institute of Public Health conducts research in various fields, in order to submit policy proposals to the Ministry of Health, Labour and Welfare, to present scientific evidence for the Ministry to formulate policies, and to provide scientific assessment on policies. The Institute also to carry out education and training of the personnel engaging in the works of public health, environmental hygiene and social welfare, and to conduct research in these areas, with a view of developing health and medical services. |
| **International best practice benchmark(s)**  AUSTRALIA: The Victorian Health Promotion Foundation (VicHealth) was the world’s first health promotion foundation, established by the Victorian Parliament as part of the Tobacco Act of 1987 (for the first 10 years through a hypothecated tobacco tax) through which the objectives of VicHealth are stipulated. VicHealth continues to maintain bipartisan support. |
| **12 PLATFORMS FOR INTERACTION**:  There are coordination platforms and opportunities for synergies across government departments, levels of government, and other sectors (NGOs, private sector, and academia) such that policies and actions in food and nutrition are coherent, efficient and effective in improving food environments, population nutrition, diet-related NCDs and their related inequalities |
| **PLATF1**: There are robust coordination mechanisms across departments and levels of government (national and local) to ensure policy coherence, alignment, and integration of food, obesity and diet-related NCD prevention policies across governments |
| <Laws/Acts>  The Health Promotion Act stipulates that the related parties of the state, prefectures, municipalities, workers of health promotion program, medical institutions and other related institutions, should make best efforts with each other in an integrated manner.  <Policies>  The Nutritional improvement campaigns under the Health Japan 21 (the second term) are conducted to enhance the achievement of targets for improvement of nutrition and dietary habits: 1) Increase in percentage of individuals who eat balanced diet with staple food, main dish and side dish more than twice a day, 2) Increase in consumption of vegetables and fruits and 3) Decrease in mean salt intake. The Ministry of Health, Labour and Welfare develops effective activities in association with prefectures, cities with public health centers, special wards and municipalities. |
| **International best practice benchmark(s)**  FINLAND: The Finnish National Nutrition Council is an inter-governmental expert body under the Ministry of Agriculture and Forestry with advisory, coordinating and monitoring functions. It is composed of representatives elected for three-year terms from government authorities dealing with nutrition, food safety, health promotion, catering, food industry, trade and agriculture.  MALTA: Based on the Healthy Lifestyle Promotion and Care of NCDs Act (2016), Malta established an inter-ministerial Advisory Council on Healthy Lifestyles in August 2016 to advise the Minister of Health on any matter related to healthy lifestyles. In particular, the Advisory Council advises on a life course approach to physical activity and nutrition, and on policies, action plans and regulations intended to reduce the occurrence of NCDs. The prime minister appoints the chair and the secretary of the Advisory Council, while the ministers of education, health, finance, social policy, sports, local government, and home affairs appoint one member each.  PACIFIC COUNTRIES: In 2014, the Pacifici Non-Communicable Disease Partnership was established to encourage a multi-sector approach to prevent and control non-communicable diseases. The partnership includes Pacific Island Forum Leaders, Pacific Ministers of Health, Pacific Islands Permanent Missions at the United Nations, Pacific Island Countries and Territories, Secretariat of the Pacific Community, World Health Organization, United Nations Development Programme, World Bank, Australia Department of Foreign Affairs and Trade, New Zealand Aid Programme, US Department of State, Pacific Island Health Officers’ Association and the NCD Alliance. The partnership aims to strengthen and coordinate capacity and expertise to support Pacific Island countries achieve globally agreed NCD targets and implement the Pacific Islands NCD Roadmap. |
| **PLATF2**: There are formal platforms between government and the commercial food sector to implement healthy food policies |
| <Laws/Acts>  The Basic Act on Shokuiku (Food and Nutrition Education) stipulates that those firms and associated organizations engaged in food manufacturing, processing, distribution, sale and provision should, through their operation, take initiative to promote food and nutrition education positively. The Act also recommends the organizations to make their best efforts to cooperate with governments including local areas and to enhance policies and activities on food and nutrition education.  <Policies>  1) The Smart Life Project is a national movement with the slogan “Let's extend healthy life expectancy” conducted by The Ministry of Health, Labour and Welfare. The Ministry promotes the project in cooperation with industries, associations and municipal governments, and their activities are introduced on the official website.  2) The Japan Food Service Association, a general incorporated association, established with the approval of the Ministry of Agriculture, Forestry and Fisheries, provides a forum to explore Japan’s current food and nutrition education with its member firms, by organizing seminars and presentations on various cases. |
| **International best practice benchmark(s)**  UK: The UK ‘Responsibility Deal’ was a UK government initiative to bring together food companies and non-government organisations to take steps (through voluntary pledges) to address NCDs during 2010-2015. It was chaired by the Secretary of State for Health and included senior representatives from the business community (as well as NGOs, public health organisations and local government). A number of other subgroups were responsible for driving specific programs relevant to the commercial food sector. |
| **PLATF3**: There are formal platforms for regular interactions between government and civil society on food policies and other strategies to improve population nutrition |
| <Laws/Acts>  The Administrative Procedure Act stipulates that Organs Establishing Administrative Orders, etc. should publicize contents and related information to be legislated in advance, and inform the public of where to submit opinions (including information) and the deadline, allowing sufficient time to seek public comments from a wider audience.  <Policies>  1) In the Consumer Affairs Agency, risk communications are conducted, that is to exchange information and opinions among consumers, business operators, government institutions, etc., in the process of risk analysis, including risk assessment and management.  2) The National Convention for Shokuiku Promotion is a nationwide core event organized in June every year during the month of food and nutrition education period, when the Expansion of Campaign Promoting Shokuiku (Food and Nutrition Education) is implemented intensively and effectively. In the convention, various exhibitions and events on food and nutrition education are organized by the state, local governments and related organizations, which are open to the public for free. |
| **International best practice benchmark(s)**  BRAZIL: The National Council of Food and Nutrition Security (CONSEA) is a body made up of civil society and government representatives, which advises the President’s office on matters involving food and nutrition security. CONSEA is made up from one-third government and two-thirds non-government executives and workers. It has special powers. It is housed in and reports to the office of the president of the republic. It is responsible for formulating and proposing public policies whose purpose is to guarantee the human right to healthy and adequate food. There are also CONSEAs at state and municipal levels that deal with specific issues, also responsible for organising CONSEA conferences at their levels. CONSEAs are charged to represent Brazilian social, regional, racial and cultural diversity at municipal, state or national level. The elected politicians in Brazil's parliament formally have the power to challenge and even overturn proposals made by CONSEA. In practice it is most unlikely that any Brazilian government whether of the left or right would wish to do so, partly because of the constitutional status of the CONSEA system, and also because, being so carefully representative of all sectors and levels of society, it remains strong and popular. |
| **PLATF4**: The government leads a broad, coherent, effective, integrated and sustainable systems-based approach with local organisations to improve the healthiness of food environments at a national level |
| <Laws/Acts>  The Health Promotion Act stipulates that the state, prefectures, municipalities, practitioners of health promotion, medical institutions should make their best efforts to liaise and cooperate with each other for health promotion in an integrated manner.  <Policies>  1) The Ministry of Health, Labour and Welfare published a FY2018 version of a standardized Health Checkups and Healthcare Advice Programs. With regard to setting up a system to review the contents of health checkups and healthcare advice incorporating the latest knowledge, the program shows roles to be played by the government, related associations, prefectures and municipalities respectively, and indicates the necessity to gather data continuously on health checkups and healthcare advice by respective parties.  2) The Prefectural-level working group in charge of Food and Nutrition Action is organized by the Ministry of Health, Labour and Welfare, where person-in-charge from respective prefectures discuss trends and issues on nutrition policies. |
| **International best practice benchmark(s)**  AUSTRALIA: Healthy together Victoria in Australia aims to improve people's health where they live, learn, work and play. It focuses on addressing the underlying causes of poor health in children's settings, workplaces and communities by encouraging healthy eating and physical activity, and reducing smoking and harmful alcohol use. Healthy Together Victoria incorporates policies and strategies to support good health across Victoria, as well as locally-led Healthy Together Communities. The initiative was originally jointly funded by the State Government of Victoria and the Australian Government through the National Partnership Agreement on Preventive Health. It is unclear at this stage whether funding for Healthy Together Victoria will continue or not.  NEW ZEALAND: Healthy Families NZ is a large-scale initiative that brings community leadership together in a united effort for better health. It aims to improve people’s health where they live, learn, work and play, in order to prevent chronic disease. Led by the Ministry of Health, the initiative will focus on ten locations in New Zealand in the first instance. It has the potential to impact the lives of over a million New Zealanders. The Government has allocated $40 million over four years to support Healthy Families NZ. |
| **13 HEALTH IN ALL POLICIES**:  Processes are in place to ensure policy coherence and alignment, and that population health impacts are explicitly considered in the development of government policies |
| **HIAP1**: There are processes in place to ensure that population nutrition, health outcomes and reducing health inequalities or health impacts in vulnerable populations are considered and prioritised in the development of all government policies relating to food |
| <Laws/Acts>  The Health Promotion Act cites seven basic policies. On national nutrition and health, it demonstrates basic polices in Item 1 of Article 10, Basic items relating to surveys of public health and nutrition and other surveys or research relating to health promotion and Items relating to the dissemination of correct awareness relating to diet, exercise, rest, alcohol, smoking, maintenance of dental health and other lifestyles. No clear reference is made to any health disparities. However, this Act shows basic policies to promote public health in a comprehensive way.  <Policies>  The Ministry of Health, Labour and Welfare evaluates the Ministry’s administration-related policies based on four basic pillars and reflects the results of the evaluation in their policies properly. Through these efforts, the Ministry tries to administer policies and services based on the viewpoints of nationals, and hence, to improve the life of the citizens. |
| **International best practice benchmark(s)**  SLOVENIA: Slovenia undertook a Health Impact Assessment (HIA) in relation to agricultural policy at the national level. This was the first time that the health effects of an agricultural policy were assessed at the country level. The HIA followed a six-stage process: policy analysis; rapid appraisal workshops with stakeholders from a range of backgrounds; review of research evidence relevant to the agricultural policy; analysis of Slovenian data for key health-related indicators; a report on the findings to a key cross-government group; and evaluation. |
| **HIAP2**: There are processes (e.g. health impact assessments) to assess and consider health impacts during the development of other non-food policies |
| <Laws/Acts>  1) The Cabinet established the Act on the Arrangement of Related Acts to Promote Work Style Reform.  2) The Basic Environmental Act aimed to ensure healthy and cultured living for both the present and future generations of the nation with prescribing the basic policy considerations for environmental conservation, such as formulation of Basic Environment Plan, establishing Environmental Quality Standards, Formulation of Environmental Pollution Control Program and Promotion of Environmental Impact Assessment.  <Policies>  1) The Guidelines that employer must endeavor to take the necessary measures to properly handle information on the physical and mental condition of workers, as provided by the Ministry of Health, Labour and Welfare, states that the management should secure health of workers, and properly collect and utilize health information, in a civil case to take safety measures for the employees.  2) The Basic Environmental Plan, presented by the Ministry of the Environment, stipulates an integrated and long-term policy outline for the whole government to pursue conservation of the environment, in accordance with Article 15 of the Basic Environmental Act. The Plan has been reviewed several times since 1994, the current one being the 5^th^.  3) In order to solve the issue of disadvantaged shoppers whose daily shopping opportunities are limited, the Ministry of Economy, Trade and Industry publicized the Second edition of Shopping Accessibility Aid Manual and publishes a best practice for public awareness, so that the private sector, municipalities and citizens can cooperate with each other. |
| **International best practice benchmark(s)**  AUSTRALIA: Established in 2007, the successful implementation of Health in All Policies (HiAP) in South Australia has been supported by a high level mandate from central government, an overarching framework which is supportive of a diverse program of work, a commitment to work collaboratively and in partnership across agencies, and a strong evaluation process. The government has established a dedicated HiAP team within South Australia Health to build workforce capacity and support Health lens Analysis projects. Since 2007, the South Australian HiAP approach has evolved to remain relevant in a changing context. However, the purpose and core principles of the approach remain unchanged. There have been five phases to the work of HiAP in South Australia between 2007 and 2016: 1) Prove concept and practice emerges (2007-2008), 2) Establish and apply methodology (2008-2009), 3) Consolidate and grow (2009-2013), 4) Adapt and review (2014) and 5) Strengthen and systematise (2015-2016).  FINLAND: Finland worked towards a Health in All Policies (HiAP) approach over the past four decades. In the early 1970s, improving public health became a political priority, and the need to influence key determinants of health through sectors beyond the health sector became evident. The work began with policy on nutrition, smoking and accident prevention. Finland adopted HiAP as the health theme for its EU Presidency in 2006. |

All of national references were Japanese and confirmed the validity and accuracy of the evidence of food-environment policies from four experts in 2019.

The international benchmarks for the best practice indicators were referenced by the World Cancer Research Fund NOURISHING database and from international experts of INFORMAS.
