# Supplementary material for: An assessment of implementation gaps and priority recommendations on food environment policies: the Healthy Food Environment Policy Index in Japan
Source: Public Health Nutr. 2021 Dec 20;25(6):1720–32. doi: 10.1017/S1368980021004900 (PMC9991651; doi:10.1017/S1368980021004900)
Supplement: Supplementary file 1 [file S1368980021004900sup.zip › S1368980021004900sup002.docx]

Supplemental File 2: The policy implementation levels against international best practice

| Indicators | n | Mean | SD | Mean (SD), Implementation level^a^ |
| --- | --- | --- | --- | --- |
| **Policy component** |  |  |  |  |
| COMP1: Processed food composition | 66 | 34.8 | 20.8 | 32.6 (19.0)  Low |
| COMP2: Out-of-home meal composition | 66 | 30.3 | 16.9 |  |
| LABEL1: Nutrient declarations on labels | 66 | 60.0 | 24.8 | 46.3 (26.2)  Low |
| LABEL2: Health and nutrient claims on labels | 66 | 63.6 | 24.2 |  |
| LABEL3: Front-of-pack labels | 66 | 26.7 | 11.8 |  |
| LABEL4: Menu board labelling | 66 | 34.8 | 20.5 |  |
| PROMO1: Broadcast advertising | 66 | 27.6 | 16.7 | 28.6 (16.8)  Low |
| PROMO2: Non-broadcast advertising | 66 | 31.8 | 18.6 |  |
| PROMO3: Advertising in child settings | 66 | 26.4 | 14.5 |  |
| PRICES1: Taxes or levies on healthy foods | 65 | 39.4 | 19.0 | 36.0 (20.8)  Low |
| PRICES2: Taxes or levies on unhealthy foods | 66 | 23.9 | 10.7 |  |
| PRICES3: Subsidies on foods | 66 | 51.5 | 24.6 |  |
| PRICES4: Food-related income support programmes | 66 | 29.4 | 15.0 |  |
| PROV1: School food standards | 66 | 70.3 | 23.1 | 62.6 (25.7)  Medium |
| PROV2: Public sector setting food standards | 66 | 46.4 | 24.6 |  |
| PROV3: Training for schools and public sector settings | 66 | 71.5 | 23.3 |  |
| PROV4: Workplace food provision | 66 | 62.1 | 24.3 |  |
| RETAIL1: Planning policies to limit take-aways | 65 | 26.8 | 14.7 | 26.7 (14.2)  Low |
| RETAIL2: Planning policies to encourage fruit & veg | 66 | 30.6 | 17.3 |  |
| RETAIL3: In-store availability of healthy foods | 66 | 26.1 | 13.6 |  |
| RETAIL4: Food service promotion of healthy foods | 66 | 23.3 | 9.7 |  |
| TRADE1: Risk impact assessments in negotiation | 66 | 60.9 | 26.6 | 55.0 (25.6)  Medium |
| TRADE2: Investment management | 65 | 48.9 | 23.2 |  |
| **Infrastructure-support component** |  |  |  |  |
| LEAD1: Political support (Cabinet level) | 66 | 60.9 | 22.9 | 65.8 (24.9)  Medium |
| LEAD2: Population intake targets established | 66 | 75.2 | 23.5 |  |
| LEAD3: Dietary guidelines established | 66 | 77.3 | 20.4 |  |
| LEAD4: Comprehensive implementation plan | 66 | 54.2 | 24.4 |  |
| LEAD5: Inequalities reduced | 66 | 61.2 | 25.3 |  |
| GOVER1: Restriction of commercial influences | 64 | 70.0 | 26.9 | 74.3 (24.3)  Medium |
| GOVER2: Evidence-based policies | 66 | 76.7 | 23.2 |  |
| GOVER3: Transparency in policies | 65 | 68.0 | 23.4 |  |
| GOVER4: Access to information and key documents | 66 | 82.4 | 21.2 |  |
| MONIT1: Monitoring of food environments | 66 | 63.6 | 23.8 | 79.3 (22.5)  High |
| MONIT2: Monitoring of nutrition status and population intakes | 66 | 81.5 | 21.7 |  |
| MONIT3: Monitoring of overweight and obesity | 66 | 90.9 | 16.5 |  |
| MONIT4: Monitoring of NCD risk factors | 66 | 84.8 | 18.2 |  |
| MONIT5: Evaluations of major programmes and policies | 66 | 77.0 | 22.0 |  |
| MONIT6: Monitoring of inequalities | 66 | 77.9 | 22.8 |  |
| FUND1: Funding for population nutrition | 65 | 66.2 | 23.7 | 72.0 (23.7)  Medium |
| FUND2: Funding for research | 66 | 68.8 | 21.4 |  |
| FUND3: Health promotion agency with secure funding | 66 | 77.9 | 24.6 |  |
| PLATF1: Coordination mechanisms across government | 65 | 67.7 | 22.0 | 67.3 (22.0)  Medium |
| PLATF2: Platforms with commercial sector | 66 | 66.1 | 22.7 |  |
| PLATF3: Platforms with civil society | 66 | 68.5 | 21.9 |  |
| PLATF4: Systems-based approach | 66 | 67.0 | 21.8 |  |
| HIAP1: Processes to reduce inequalities | 66 | 68.2 | 24.0 | 67.8 (23.3)  Medium |
| HIAP2: Processes to assess health impacts | 65 | 67.4 | 22.8 |  |

SD, standard deviation:, COMP: Food composition, LABEL: Food labelling, PROMO: Food promotion, PRICES: Food prices, PROV: Food provision, RETAIL: Food retail, TRADE: Food trade and Investment, LEAD: Leadership, GOVER: Governance, MONIT: Monitoring and intelligence, FUND: Funding and resources, PLATF: Platforms for interaction, HIAP: Health in all policies

^a^ The implementation level was categorized ≤ 25% as “very low if any”, 26 to 50% as “low”, 51 to 75% as “medium”, and >75% “High”.
